# ^1^H NMR metabolomics analysis of oil palm stem tissue infected by *Ganoderma boninense* based on field severity Indices

**DOI:** 10.1038/s41598-022-25450-5

**Published:** 2022-12-06

**Authors:** Adi Pancoro, Elfina Karima, Ardha Apriyanto, Yunus Effendi

**Affiliations:** 1 School of Life Sciences and Technology, Bandung Institute of Technology, Bandung, 40132 Indonesia; 2 Astra Agro Lestari Tbk, Research and Development, Jakarta, 13920 Indonesia; 3 Biological Science Department, Al-Azhar Indonesia University, Jakarta, 12110 Indonesia

**Keywords:** Metabolomics, Metabolomics, Bioanalytical chemistry, Biotic

## Abstract

Basal stem rot disease (BSR) caused by *G. boninense* affects most oil palm plants in Southeast Asia. This disease can be fatal to palm oil production. BSR shows no signs on the tree in the early stages of infection. Therefore, it is essential to find an approach that can detect BSR disease in oil palm, especially at any level of disease severity in the field. This study aims to identify biomarkers of BSR disease in oil palm stem tissue based on various disease severity indices in the field using ^1^H NMR-based metabolomics analysis. The crude extract of oil palm stem tissue with four disease severity indices was analyzed by ^1^H NMR metabolomics. Approximately 90 metabolites from oil palm stem tissue were identified.Twenty of these were identified as metabolites that significantly differentiated the four disease severity indices. These metabolites include the organic acid group, the carbohydrate group, the organoheterocyclic compound group, and the benzoid group. In addition, different tentative biomarkers for different disease severity indices were also identified. These tentative biomarkers consist of groups of organic acids, carbohydrates, organoheterocyclic compounds, nitrogenous organic compounds, and benzene. There are five pathways in oil palm that are potentially affected by BSR disease.

## Introduction

Palm oil is the world's most consumed oil produced by oil palms (*Elaeis guineensis* Jacq.). This oil consumption can reach almost 3 billion people in 150 countries, including China, India, Indonesia, and the European Union^1^. Indonesia is one of the largest palm oil producers globally^2,3^. However, the palm oil industry faces a threat from a disease called basal stem rot (BSR), which can alleviate palm oil production by 50–80%^4^. BSR disease is caused by a white-rot fungus that is able to break down the lignin content of wood, exposing the white cellulose content of wood^5^. Several studies have reported on the mechanism of BSR infection^6–9^. This causes the same symptoms in oil palms when plants are stressed by water scarcity and malnutrition^6^.

The severity of BSR in the field is divided into 4 different disease severity indices. This classification is based on the visual analysis of observers commonly used by field technicians in monitoring BSR disease^7^. The Disease Severity Index includes Index 1 (healthy) with no symptoms, Index 2 (moderately healthy) with symptoms of leaf colour changing to yellowish to faint, Index 3 (moderately severe) with symptoms on leaves turning yellowish to faint discolour and young stalks that do not open, and Index 4 (severe) indicated by symptoms like Index 3 but associated with fruiting bodies of fungi growing on oil palm stems. The difference in appearance between healthy trees and infected trees is caused by damage to the internal tissues that can disrupt photosynthetic activity and therefore affect the deterioration of the tree's physical condition^8^. Disturbance of new leaf growth and undeveloped leaves and racemes in severe cases can also occur due to nutrient deficiencies^9^. However, based on numerous reports, physical symptoms will only appear as early as 2 months after the inoculation period^10^. This is believed to be caused by a fungus growing inside the tree before the physical signs of infection appear^11^. The difficulty of early detection of BSR disease has become a reference point for various studies to find the best method to diagnose BSR, although there have not been reasonable results^12^.

Metabolomics is a potential approach for the early detection of BSR disease by identifying metabolite changes in *G. boninense*-infected trees. This approach aims to detect and quantify metabolic changes in a biological system under a stimulus, both internal stimuli, such as gene changes, and external stimuli, such as pathogen infection^13^. Previous studies have reported the occurrence of metabolic changes in oil palms caused by BSR disease^12,14,15^. Metabolomics profiling utilizing gas chromatography-mass spectrometry (GC-MS) on noninfected and *G. boninense-*infected oil palm root tissue showed that steroidal compounds and fatty acid derivatives were more abundant in diseased oil palm roots than in healthy controls^15^. A previous study reported that fatty acids and their methyl esters could act as antimicrobial agents against pathogens^16^ whereas high concentrations of steroidal compounds could act as plant defence metabolites^17^ in which the plant defence response could initiate signalling events in the sterol biosynthesis pathway that lead to stigmasterol induction^18^. Metabolomics analysis of leaf tissue using ^1^H nuclear magnetic resonance (NMR)^14^ also shows significant differences in which infected leaves had higher relative abundances of ﻿choline, asparagine, alanine, succinic acid, gallic acid, and other metabolites. Metabolite comparisons of oil palm with different susceptibilities to *G. boninense* have also been performed using metabolomics approaches^19,20^. The study of metabolite comparisons in root tissues of parental palms that are partially tolerant and susceptible to *G. boninense* has successfully identified 9 metabolites with a range of plant sugar and phenolic derivatives using liquid chromatography-mass spectrometry (LC–MS)^20^. Metabolite change analysis based on field severity indices has been performed using high-performance liquid chromatography (HPLC)^7^ and liquid chromatography coupled with time-of-flight mass spectrometry (LC-Q/TOF-MS)^21^. Another study showed that there was a strong correlation between oil palm disease severity indices and ergosterol relative concentration levels that were analyzed using HPLC^7^.

Network representations of biological systems are widely used in modern biology to study systemic connections within biological parts^22,23^. In metabolic networks, the reconstruction of networks from metabolite profiling data would not only be useful for the visualization of metabolic pathways but also for analysing the global structure of datasets^24^. This network contains a collection of nodes that represent metabolites and edges that represent relationships between metabolites^25^. Debiased sparse partial correlation (DSPC) is an algorithm based on the recently proposed de-sparsified graphical lasso modelling procedure^26^ that can provide partial correlation coefficients and *p*-values for every metabolic feature pair in a dataset and reconstruct its graphical model. Unlike widely used correlation networks, partial correlation networks can distinguish between direct and indirect correlations and provide insights into the dependence structure between metabolites^27^.

NMR spectroscopy is widely used for metabolomics analysis due to its high reproducibility and simple sample preparation steps and its quantitative ability^28–30^. Previous studies have successfully identified biomarkers of BSR disease in diseased oil palm leaves from infected seedlings using NMR-based metabolomics analysis as previously discussed^12,14^. To date, no studies have reported metabolomics analysis of oil palm stem tissue under various disease severity indices in the field using ^1^H NMR as well as the reconstruction of metabolomics networks using the correlation-based network analysis biased sparse partial correlation (DSPC) approach. The main objective of this study was to identify biomarkers of basal stem rot (BSR) disease in oil palm stem tissue with 4 different disease severity indices using metabolomic analysis as well as reconstruction of the metabolomic network possibly linked to *G. boninense* infection in oil palms. The results of this study are expected to be used as a diagnostic tool for basal stem rot at various disease severity levels and as a reference for further genomic and transcriptomic studies.

## Results

### NMR profiles of Ganoderma-infected oil palm stem tissue in 4 severity indices

**Figure 1:**
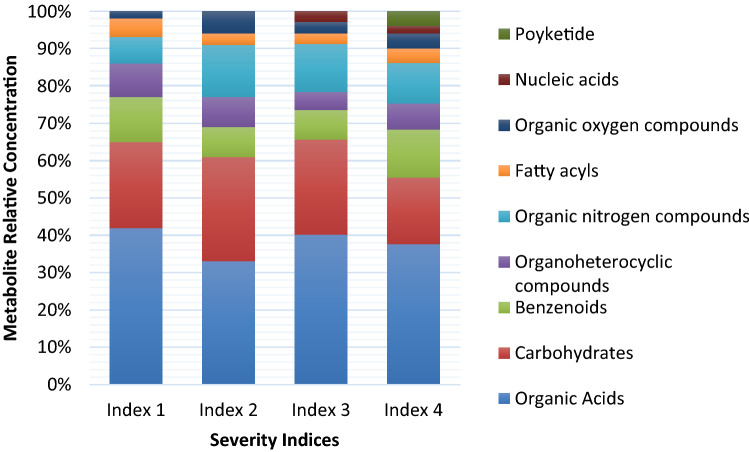
Metabolite profile of oil palm with 4 different severity indices. Each compound category is represented with relative percentages.

Based on the results of ^1^H NMR analysis, 90 types of metabolites from 4 different levels of disease severity were identified. These are the metabolites those were characterized by comparing spectra obtained and ASICS database.These metabolite consists of 65 metabolites for severity index 1, 51 for severity index 2, and 72 for each severity index 3 and 4 while the exclusive metabolites for each index are 63 for index 1, 48 for index 2, 63 for index 3 and 4 (Supplementary Table 1). The enrichment analysis step was applied to classify these metabolites into 9 main metabolite classes of organic compounds: organic acids, carbohydrates, benzenoids, organoheterocyclic compounds, organic nitrogen compounds, fatty acyls, organic oxygen compounds, nucleic acids, and polyketides. We classified the unclassified metabolites based on the metabolite classes in the HMDB. The analysis results showed that organic acids (including amino acids) and carbohydrate derivatives were the class of compounds with the highest percentage in all disease severity indices (Figure 1). Index 1 has the highest relative percentage of organic acids (43%) compared to other indices, while Index 2 has the lowest relative percentage of organic acids (33%). Index 4 has the lowest relative percentage of carbohydrates (18%), while other indices have a relatively higher percentage of carbohydrates (23%, 28%, and 26% for Indices 1, 2, and 3, respectively). The class of fatty acyls and benzenoids (including phenolic compounds) was also present in all indices, although the percentage was not as high as that of organic acids. Benzenoids had the highest relative percentage in Indices 1 and 4 and the lowest relative percentage in Indices 2 and 3. Two organic compounds are present in Indices 3 and 4 that are not present in Indices 1 and 2: polyketides for Index 3 and polyketides and nucleic acids for Index 4. Differences in metabolites detected in oil palm stem tissues with different disease severity indices were then analysed using multivariate statistical analysis.

### Multivariate data analysis (MVDA)

**Figure 2:**
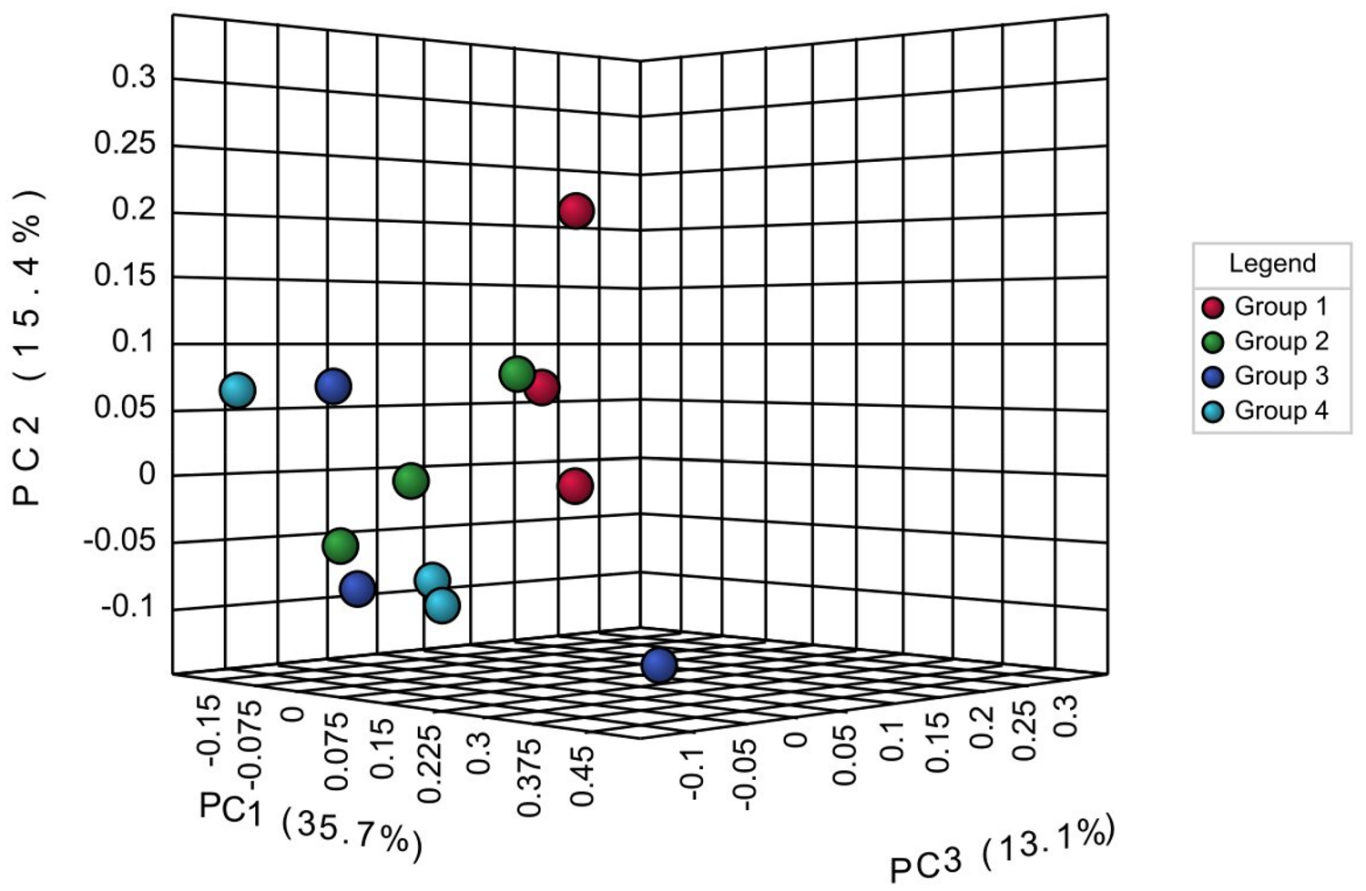
The results of 3D PCA visualization against all disease severity indices to see differences in metabolite profiles in each severity condition.

The identification of metabolite profiles and significant compounds in this research was made using multivariate data analysis. PCA was performed to classify the sample characteristics and analyse the metabolites that contributed to the variation in the data^14^. PCA was performed for all disease severity indices (Figure 2) to identify differences in total metabolite profiles, and paired PCA analysis at indices 1–2, 1–3, and 1–4 (Supplementary Figure 1) was performed to determine the differences in the metabolite profile between the health indices and various other disease severity levels.

The paired PCA analysis should identify potential differences in metabolites between 2 states (healthy and different disease severity levels) for their application as biomarkers in the early diagnosis of BSR diseases. In Figure 2, it is shown that there are differences in metabolite profiles between different disease severity levels, although the 3D PCA visualization is not fully separated. In this analysis, the cumulative value of the two initial principal components (PC1 and PC2) is 51.1% of the total variation. These 4 disease severity groups separated mainly in the PC1 direction, which had an overall variation of 35.7% of the total NMR data. Pairwise PCA between indices 1 and 2 shows a distinction between the two disease severity states, although there is still overlap between the two groups, with the cumulative total of the principal components being 69.8% (Supplementary Figure 1). The two groups are also well separated towards PC1, where the total variation value is 42.5%. In the paired PCA analysis between indices 1–3 and 1–4, the differences in metabolite profiles were quite clear, although there was still overlap between groups 1 and 4. The cumulative values ​​of PC1 and PC2 for each pair were 72.1% and 56.3%, respectively. The separation between these two groups also occurs well towards PC1, where the variation value is 51.3% for index 1–3 and 32.9% for index 1–4. From the overall PCA analysis, each disease severity index has a compound profile that is quite different from each other. The paired PCA analysis was able to show that there was a clear difference between the healthy plant (index 1) and other severity indices, so there is potential for the identification of BSR disease tentative biomarkers for early detection purposes through this study.

**Figure 3:**
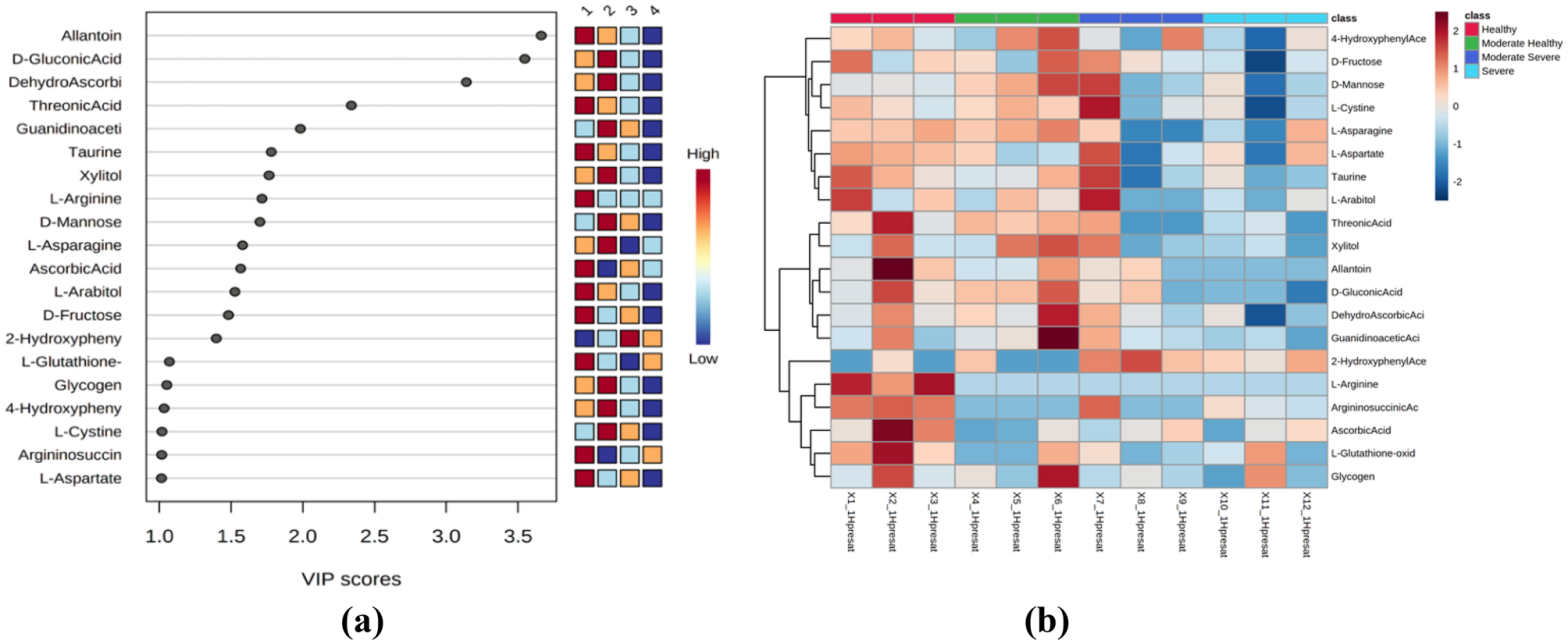
(**a**) The results of the analysis of significant compounds using PLS-DA analysis on all disease severity indices. (**b**) Heatmap visualization results on all disease severity levels. The red color on the heatmap indicates a relatively high concentration, and the blue color indicates a relatively low concentration. The x-axis in the figure represents each sample in this analysis, the top x-axis represents disease severity index class, the right y-axis represents each identified compound, and the left y-axis represents the grouping of compounds by dendrograms.

PLS-DA^31,32^ analysis was used to distinguish significant metabolites between all disease severity indices, while OPLS-DA^33^ analysis was performed to determine significant metabolites between the two disease severity indices, namely, index 1 and the other severity indices (Figure 3a). Based on the results of the PLS-DA analysis, 20 metabolites that could significantly differentiate the four disease severity indices were identified (VIP score > 1.0; R2 = 0.83; Q2 = 0.15). Lists of metabolites can be found in Table 3. These metabolites include organic acid, carbohydrate, organoheterocyclic compound, and benzenoid groups. The relative concentration of each compound is represented by red for the highest relative concentration and blue for the lowest relative concentration. Based on the analysis, 31, 25 and 33 significant metabolites were identified from OPLS-DA analysis for indexes 1–2, 1–3, and 1–4, respectively (VIP score > 1.0) (Supplementary Figure 1). Some organic acid compounds, such as taurine and threonic acid, have relatively high concentrations at index 1 but very low concentrations at index 4. Carbohydrate groups such as L-arabitol and D-fructose as well as organoheterocyclic compounds such as allantoin had the highest concentration at index 1 and decreased at the other indices. This is in complete contrast to the research of Isha et al.,^12^ where in *G. boninense*-infected leaves, the relative concentration of D-fructose actually increased compared to healthy leaves. This may indicate that in infected stem tissue, some of the carbon that makes up carbohydrate compounds is transferred to secondary metabolism^14^. Carbohydrates can also act as an essential energy source in the biosynthesis of secondary metabolitesClick or tap here to enter text., so the relative concentration decreases when the disease severity index increases. However, for other carbohydrate-derived compounds, such as D-gluconic acid, xylitol, and D-mannose, the relative concentration at index 2 was actually higher than at index 1. This may be because these compounds act as signalling molecules in biotic stress^34^. The accumulation of these compounds may also be related to their role as energy sources for pathogens^35^.

Heat map analysis was performed to visualize the concentration of each metabolite at different indices (Figure 3b). Heat map analysis was performed for all disease severity indices. Based on the dendrograms of all heat map combinations, metabolites are classified into two main groups, namely, metabolites with high concentrations in certain severity indices and metabolites with low concentrations in other severity indices. For example, in the index 1-2-3-4 heat map visualization, there are two groups of compounds that tend to have relatively high concentrations at index 1 but decrease at the next index and groups of compounds with relatively high concentrations at both indices 1 and 2 but decrease in the next index. The first group of compounds consisted of L-arginine, ascorbic acid, oxidized L-glutathione and 2-hydroxyphenylacetic acid. The next group includes allantoin, D-gluconic acid, guanidino acetic acid and other compounds. The heatmap also shows that some sugar compounds, such as D-mannose, L-arabitol, and D-fructose, tend to have a high concentration on Disease Severity Index 3 and 4 compared to Index 1 (Healthy). This might indicate that these compounds have the potential to act as signalling molecules under biotic stress^34^ as well as an energy source for pathogens^35^.

### Biomarker analysis

Biomarkers can be interpreted as biological indicators that can indicate the presence, absence or status of a disease and ideally have sensitive and specific properties^36^. The identification of tentative biomarkers on *G. boninense*-infected oil palm stem tissue at different disease severity indices was performed. This tentative biomarker is expected to be used as a diagnostic tool for basal stem rot disease in oil palm at different disease stages. The methods used in this analysis are OPLS-DA and receiver operating characteristics (ROC). OPLS-DA was used as a method to classify significant compounds with a VIP cutoff > 1.0^37^ while the ROC curve method was used to assess the diagnostic power of biomarkers^36^. From the results of the analysis in this study, there were 12 disease tentative biomarkers for severity index 2, 12 disease biomarkers for severity index 3, and 11 disease biomarkers for severity index 4. These tentative biomarkers consisted of groups of organic acids, carbohydrates and organoheterocyclic compounds, organic nitrogen compounds, and benzene. The table for the list of tentative biomarkers for disease severity indices 2, 3, and 4 compared to healthy trees is shown in Table 1.

**Table 1:** List of biomarkers of *G.* boninense-infected oil palm stem tissue and their VIP scores obtained by OPLS-DA analysis at severity indices 2, 3, and 4 compared to index 1.

No.	Metabolite	VIP score of tentative biomarker indices compared to index 1		
		2	3	4
1	Threonic Acid	1.25*	1.24	2.05*
2	trans-4-Hydroxy-L-Proline	1.09*	1.20	0.75
3	L-Proline	1.70*	0.58	1.68
4	L-Arginine	1.55*	–	0.91
5	L-Glutathione-oxidized	1.36*	1.32	1.55*
6	L-Ornithine	1.63*	–	0.95
7	D-Fucose	1.34*	0.42	0.22
8	L-Citrulline	1.72*	1.22	1.99*
9	Argininosuccinic acid	1.58*	1.40*	1.80*
10	Dihydrothymine	1.72*	1.44*	1.52*
11	L-Carnitine	1.29*	0.64	1.02
12	Methylguanidine	1.71*	–	0.75
13	Glyceric Acid	0.93	1.74*	0.05
14	L-Asparagine	0.05	1.50*	1.51
15	L-Glutamic Acid	–	1.81*	0.06
16	Myo-Inositol	1.15	1.80*	0.51
17	3-Methyl-L-Histidine	1.21	1.43*	0.03
18	L-Tyrosine	0.02	1.85*	–
19	Phosphocholine	–	1.40*	1.42
20	Lactose	1.21	1.58*	0.97
21	Allantoin	0.68	0.09	1.53*
22	D-Gluconic acid	0.09	0.44	1.50*
23	Glycerol	0.12	1.52*	0.90
24	L-Aspartate	1.22	1.30*	1.33*
25	Sarcosine	1.17	1.72*	2.03*
26	Creatinine	–	–	1.56*
27	2-Hydroxyphenyl Acetic Acid	0.72	1.30	1.57*

*Indicates metabolite with AUC Score = 1 which is the potential candidate of biomarker for that index.

Although they have some similarities in terms of groups of organic compounds, both of which are included in the group of fatty acid derivatives, sugars and derivatives of phenolic compounds, there are no similarities between the tentative biomarker identification results between this study and earlier research on biomarker identification using GC–MS^15^ and LC–MS^20^. Differences in biomarkers were also found when comparing the results of this study with the results of identifying biomarkers on *G. boninense*-infected oil palm leaves using NMR^12,14^. These differences can be caused by measuring instruments or the analyzed tissue differences. Despite this, the panel of connections between the tentative biomarkers in this study and previous studies shares many similarities, including many consisting of carbohydrate derivative compounds, amino acids, phenolic compounds, and other organic acid compounds.

**Figure 4:**
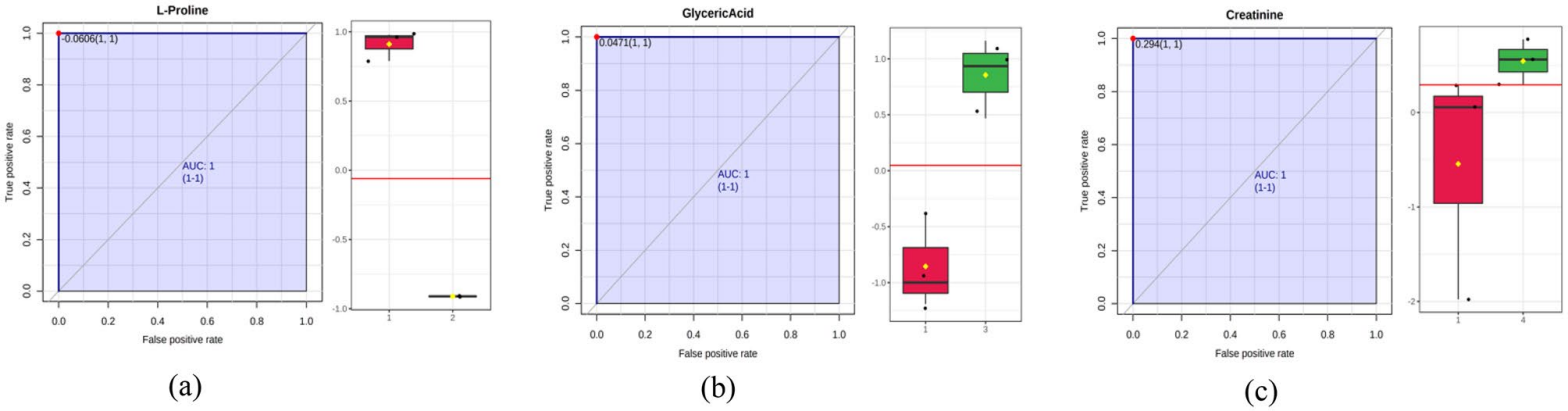
ROC curve and boxplot of (**a**) L-proline, (**b**) glyceric acid, and (**c**) creatinine as candidate biomarker compounds for Indices 2, 3, and 4, respectively.

Figure 4 shows the ROC curve and boxplots of several tentative biomarkers in this study. An AUC score of 1 indicates that the classifier is able to discriminate all values in the positive and negative classes well, so it does not allow false-positive cases to occur. Furthermore, the box plot visualization shows a significant difference between the concentrations of tentative biomarkers at two different disease severity states, in this case the concentrations of L-arginine, 2-hydroxyphenyl acetic acid, and sarcosine at disease severities of 2, 3, and 4, respectively, compared to healthy trees. Therefore, it is hoped that the tentative biomarkers identified from the results of this study can accurately predict the severity of BSR disease in oil palm stem tissue.

Based on pathway analysis using the KEGG database, there are 5 pathways in oil palm that are potentially affected by BSR disease (*p*-value < 0.05 and pathway impact > 0.1), namely, the arginine biosynthesis pathway, alanine, aspartate, and glutamate metabolism, glycine, serine, and threonine metabolism, beta-alanine metabolism, and arginine and proline metabolism (Table 2). There were 6 metabolites targeted in the analysis of the arginine biosynthetic pathway, namely, L-citrulline, L-glutamine, L-aspartate, L-argininosuccinate, L-arginine and L-ornithine. Arginine is a precursor to the biosynthesis of proline, polyamine, and NO, which play a role in the stress response of plants. Proline and NO have a regulatory function in plant development and act as signaling molecules that can mediate various responses to biotic and abiotic stress, while polyamines play a role in plant development and plant responses to stress^38^.

**Table 2:** Identification of oil palm pathways altered due to BSR disease. These pathways have *p* value < 0.05 and impact factor > 0.1.

No.	Pathway name	Match	*p* value	− log(p)	Impact
1	Arginine biosynthesis	7	3.1451E-6	5.5024	0.45632
2	Alanine, aspartate, and glutamate metabolism	5	0.001532	2.8147	0.66187
3	Glycine, serine, threonine metabolism	5	0.0097361	2.0116	0.14292
4	Beta-alanine metabolism	3	0.034777	1.4587	0.25397
5	Arginine and proline metabolism	5	0.011059	1.9563	0.39045

**Figure 5:**
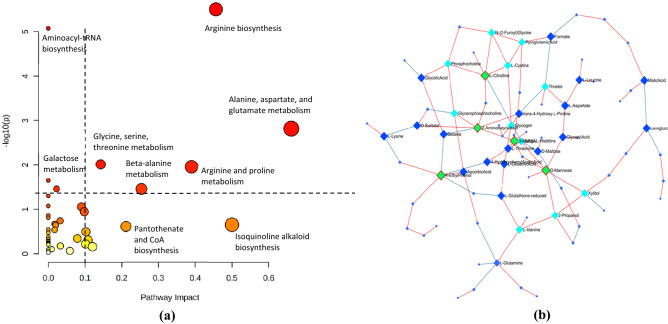
(**a**) Analysis of altered metabolic pathways based on distinguishing metabolites found in oil palm stem tissue with various BSR severity indices. (**b**) DSPC network analysis of all indices of oil palm stems infected by *G. boninense*. Red edges indicate positive correlations, and blue edges indicate negative correlations. Green nodes indicate nodes with the highest node degree and betweenness centrality.

The identification of 6 metabolites in this pathway indicates that the arginine biosynthetic pathway may be affected by BSR disease in oil palm stem tissue, where this pathway may function in response to biotic stress. In addition to the arginine biosynthetic pathway, the alanine pathway can also be affected by *G. boninense* infection. Alanine is a nonproteinogenic amino acid that plays a role in the general stress response in plants and protects plants from extreme temperature, hypoxia, drought, heavy metals, and biotic stress. Alanine can be converted into osmo-protective compounds, such as alanine-betaine, in some species and into the antioxidant homoglutathione in other species^39^. For example, in *Arabidopsis thaliana*, both drought and heat stress can increase alanine levels^40^. The next metabolic pathway that can be affected by BSR disease is the metabolism of alanine, aspartate and glutamate. Asparagine, commonly known as a nitrogen transporter in plants, is an amino acid that plays a role in the stress response from pathogens. Aspartate is a precursor of asparagine biosynthesis, the conversion of which requires the enzyme asparagine synthetase^41^. This study found six metabolites in the alanine pathway and the alanine, aspartate, and glutamate pathways. These compounds were L-aspartate, -alanine, panotenate, L-asparagine, L-aspartate, and L-arginino succinate. The other pathways that could be altered due to BSR disease are glycine, serine, and threonine metabolism and arginine and proline metabolism (Fig. 5a).

Metabolomics analysis in this research was also conducted using the Debiased Sparse Partial Correlation algorithm (DSPC)^27^. This algorithm was based on a recently proposed de-sparsified graphical lasso modeling procedure^26^, assuming that the number of actual connections between metabolites is smaller than the available sample size^42^. In this research, network analysis was conducted on the metabolites in all indices and in each index (Fig. 5b).

In metabolites from all indices, the metabolic network was constructed from 79 nodes and 100 edges. In this network, it is observed that there are 5 metabolites that have the highest node degree and betweenness centrality, namely, L-citrulline, 2-aminobutyric acid, 3-methyl-L-histidine, D-mannose, and 4-ethyl phenol. From this network, it is observed that there are various correlations between metabolites, either as a positive correlation (red edges) or a negative correlation (blue edges). At Index 1, several positive and negative correlations existed between metabolites (Supplementary Fig. 3). For instance, 4-hydroxyphenyl acetic acid has a negative correlation with D-fucose but a positive correlation with myo-inositol. In index 2 and index 4, there are also several various correlations between metabolites. However, in the index 3 network, the correlation between metabolites is mostly positive (red edges), which means that the increase in one metabolite concentration would cause an increase in other metabolites as well. Glycogen, as the compound with the highest node degree, has a positive correlation with several metabolites, such as xylitol, lactate, and sarcosine. The summary of the metabolic network from various severity indices is written in Table 3.

**Table 3:** Summary of nodes and edges of metabolic network from various indices.

Severity index	Node	Edges (Blue and red)	Green node*	Nodes with highest degree
1, 2, 3, 4	79	100	5	L-Citrulline, 2-Aminobutyricacid, 3-Methyl-L-histidine, D-Mannose, 4-Ethyl Phenol
1	60	118	1	4-Hydroxyphenyl acetic acid
2	48	101	1	4-Hydroxyphenyl acetic acid
3	70	281	1	Glycogen
4	67	117	1	D-Glucose

* nodes with highest node degree and betweenness centrality.

**Figure 6:**
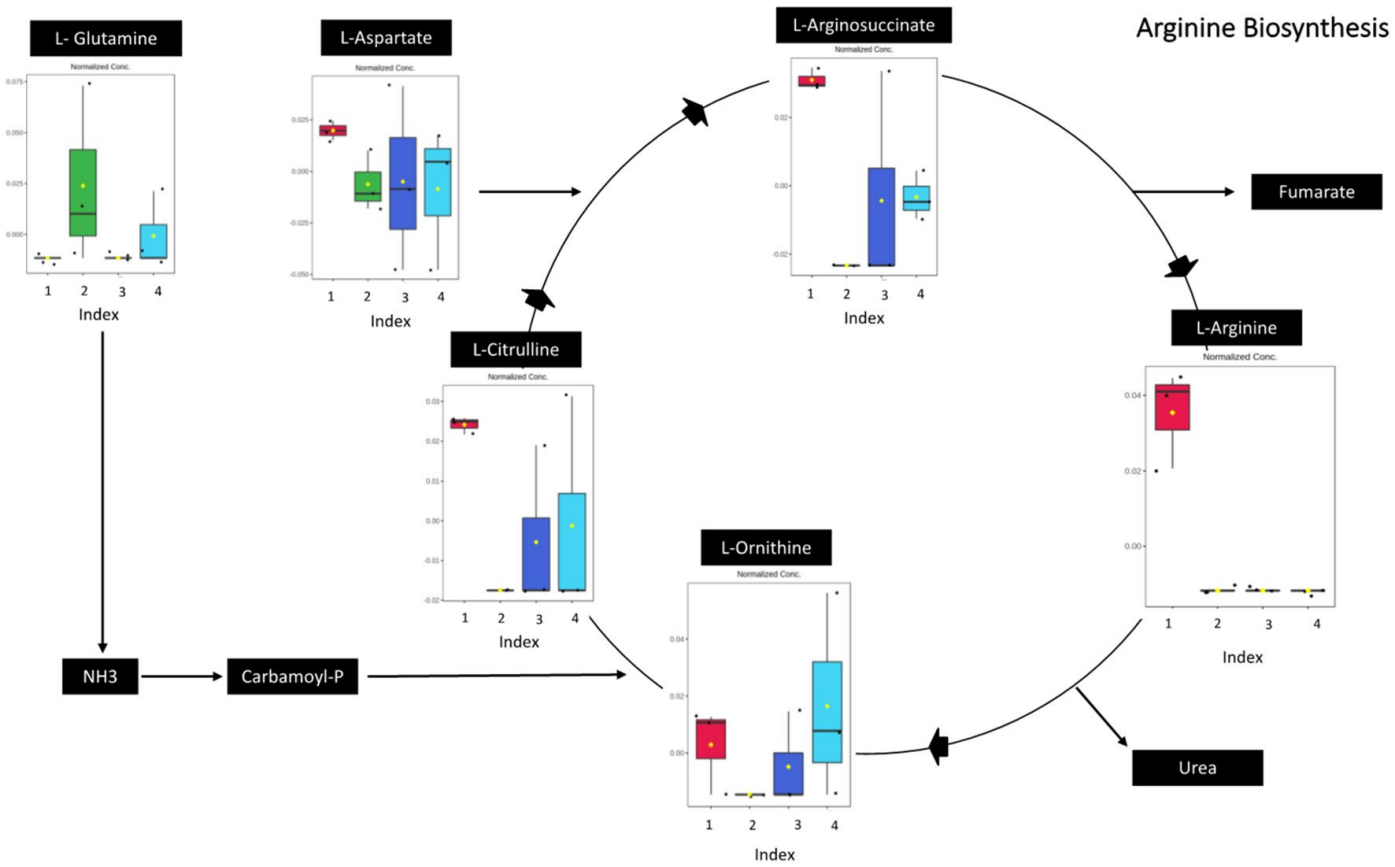
Schematic representation of 5 pathways that are potentially affected by BSR disease and relative concentrations of metabolites involved in arginine biosynthesis.

Differences in metabolite relative concentrations among all indices could be observed in the altered metabolic pathway analysis (Fig. 6). In the arginine biosynthesis pathway, L-arginine had the highest relative concentration at index 1 and the lowest at the other indices. L-Ornithine, L-citrulline, and L-arginosuccinate had the lowest relative concentrations at index 2 compared to the other indices. L-aspartate has the highest concentration at index 1 and relatively the same concentration at other indices, while L-glutamine has the lowest relative concentration at indexes 1 and 3 and the highest at indexes 2 and 4 (Supplementary Fig. 2).

## Discussion

There are several significant compounds that were successfully identified using NMR in this research. These compounds included classes of carbohydrate and derivatives, phenolic compounds, amino acids and derivatives, fat and fat-like compounds, and other organic compounds. We also compare our results those were obtained by NMR instrument with another previous metabolomics research that was obtained by GC–MS and LC–MS instrument (Table 4). Even though these comparison use the different part of plants and different instrument, but these results have showed that the infected oil palm plants have significant compound those were changed due to this condition.

**Table 4:** Results of significantly identified compounds in stem tissue metabolomics studies with NMR and compilations with previous studies using GC–MS and LC–MS.

	Metabolomics analysis of significant compounds from stem tissue using NMR	Metabolomics analysis of significant compounds from leaf tissue using NMR^12,14^	Metabolomics analysis of significant compounds from root tissue using GC–MS^15^	Metabolomics analysis of significant compounds from root tissue using LC–MS^20^
Tissue	Stem	Leaf	Root	Root
Experimental Location / Source	Field	Nursery	Nursery	Nursery
Instrumentation	NMR	NMR	GC–MS	LC–MS
**Compound Classes**				
Carbohydrates and Derivatives	D-Gluconic Acid Threonic Acid Xylitol D-Mannose L-Arabitol D-Fructose Glycogen	Xylose Sucrose Arabinose Fructose alpha-Glucose ﻿N-acetylglucosamine	No significant compounds identified	﻿Sedoheptulose
Phenolic Compounds	2-Hydroxyphenyl acetic acid 4-Hydroxyphenyl acetic acid	Caffeic Acid Gallic Acid Tyramine ﻿2,3,4-Trihydroxybenzoic acid ﻿p-Hydroxybenzoic acid Kaempferol ﻿Epicatechin ﻿p-Hydroxybenzoic Acid	No significant compounds identified	﻿Dimethoxyphenyl-O- hexose-O-pentoside ﻿Trimethoxyphenyl- O- hexose-O-pentoside ﻿Dimethoxybenzyl- O-rhamnoside ﻿Dimethoxyphenylethyl- -hexose-O-rhamnoside ﻿Pinocembrin ﻿malonylhexoside ﻿Hydroxy ﻿dimethoxybenzoyl-sulfo-﻿hexoside Procyanidin B1
Amino Acid and Derivatives	Guanidino Acetic Acid Taurine L-Arginine L-Asparagine L- Oxidized Glutathione L-Cysteine Argininosuccinic Acid L-Aspartate	Alanine 3-Aminoisobutiric Acid *N*-acetyl tyrosine *N*-acetyl tyrosine Homo cysteine Asparagine Alanine S-Sulfocysteine	No significant compounds identified	No significant compounds identified
Fat and Fat-like Compounds	No significant compounds identified	Isobutyric Acid ﻿β-Sitosterol ﻿α-Tocopherol	﻿Methyl hexadecanoate ﻿Hexadecanoic acid ﻿Methyl (9Z,12Z)-octadeca-9,12- dienoate ﻿Methyl (Z)-octadec-6-enoate ﻿Methyl octadecanoate ﻿(E)-icos-5-ene ﻿Stigmasterol ﻿Stigmast-5-en-3-ol, (3b) ﻿Ergost-5-en-3-ol, (3b)	No significant compounds identified
Other Organic Compounds	Allantoin Dehydro Ascorbic Acid Ascorbic Acid	Indol-3-Asetic Acid Choline trans-Asonetatic Acid Biotin ﻿β-Cryptoxanthin Succinic Acid Trimethyl amine ﻿2,3-Butanediol Lactic Acid	﻿2,2-Dimethoxybutane ﻿Dimethyl 2-methoxybutanedioate ﻿5-(hydroxymethyl) furan-2- carbaldehyde ﻿2,3-Dihydroxypropyl acetate ﻿2-(Hydroxymethyl)-2- nitropropane-1,3-diol ﻿Dimethyl benzene-1,4- dicarboxylate ﻿Methyl 3-(3,5-ditert-butyl-4- hydroxyphenyl) propanoate ﻿3,5-Dihydroxy-6-methyl-2,3- dihydropyran-4-one	No significant compounds identified

The analysis results showed that organic acids (including amino acids) and carbohydrate derivatives were the class of compounds with the highest percentage of all disease severity indices. Throughout the index, classes of fatty acid and benzene compounds (including phenolic compounds) were also identified in the oil palm stem tissue. The results of this identification have several similarities with the class of compounds identified in the research conducted by Isha et al.^12^. This study identified amino acids, sugars, and phenolic compounds in oil palm leaves infected with *G. boninense* by analysis using ^1^H NMR. Several classes of compounds, such as fatty acids, were also identified in research conducted on oil-diseased palm roots using GC–MS^15^. However, unlike this study, in a previous study, fatty acids were not the class of compounds with the highest percentage. Sugar compounds and phenolic compounds that belong to the benzenoid class were also found in the research conducted by Kushairi et al.^20^. In this study, 9 biomarkers identified from oil palm roots infected with G. *boninense* using LC–MS all belong to the class of sugars and phenolic compounds.

Significant compounds were defined as compounds that had statistically significant differences in relative concentrations between one disease severity index and another. There were significant differences in compound profiles between stem, leaf, and root tissues analysed by different measuring instruments. Classes of compounds with the broadest range, including carbohydrates, phenolic compounds, amino acids, fats and compounds such as fats, to various other organic compounds were identified by NMR instruments in both stem and leaf tissues. Meanwhile, the GC–MS and LC–MS instruments identified groups of fatty acid compounds and phenolic compounds in root tissue. Significant metabolites identified in this study include groups of carbohydrate compounds, phenolic compounds, amino acids, and other organic compounds. This profile is similar to the group of compounds identified in the leaves by NMR^12,14^. However, neither fatty acids nor steroidal compounds were identified as identified in roots using GC–MS and LC–MS^15,20^. Most of the compounds identified in this study were from sugar and amino acid groups. Several significant compounds from the sugar group and its derivatives were identified in this study. These compounds include D-gluconic acid, threonic acid, xylitol, D-mannose, L-arbitol, D-fructose, and glycogen. Sugars are known to act as signaling molecules in both biotic and abiotic stresses^34^. Even in several studies, it is stated that the response of plants to fungal pathogen attacks is closely related to pathways that regulate sugar levels in plant cells^43^. Based on Morkunas and Ratajczak^44^, sugar molecules are also the main substrate that provides energy and structural material for defense in plants. Sugar could increase oxidative spikes in the early stages of infection, increase cell wall lignification, stimulate flavonoid synthesis, and induce certain PR proteins.

The amino acids and their derivatives identified in this study include guanidino acetic acid, taurine, L-arginine, L-asparagine, oxidized L-glutamate, L-cysteine, argininosuccinic acid, and L-aspartic acid. Many compounds in plants that play a role in defense against pathogens are derived from amino acid precursors^45^. The amino acid asparagine identified, for example, is thought to be involved in plant stress responses associated with pathogen infection^41^. Aspartate, which was also identified in this study, was converted to asparagine by asparagine synthetase through the asparagine biosynthetic pathway in plants^46^.

2-Hydroxyphenyl acetic acid and 4-hydroxyphenyl acetic acid were the two phenolic compounds identified in this study. Phenolic compounds are phytochemical compounds that have an aromatic ring structure with at least one hydroxyl substituent on the aromatic ring. This compound is a secondary metabolite in plants and has various roles, including defense against pathogens^47^. Previous studies have extensively studied the role of phenolic compounds in defense against fungi and viral plant diseases, including the activity of phenolic monoterpene compounds against fungal pathogens in vitro^48^. Various fungitoxic mechanisms of phenolic compounds have been reported in previous studies, including distorting the integrity of the cell wall, changing cell membrane permeability, suppression of enzymes, elicitation of oxidative spikes, DNA damage, inhibition of protein synthesis, and repression of virulent genes^49–51^. The phenolic compounds identified were monocarboxylic acids, namely, acetic acid, in which one of the hydrogens in the methyl group was substituted with a hydroxyphenyl group. This compound has been identified as an antioxidant compound in several studies, such as in olive oil^52^, beer^53^, plants of the Astilbe genus^54^, and even produced by the fungus *Oidiodendron* sp. as a nematicide^55^. Although it has been identified as one of the phenolic compounds in higher plants, until now, there has been no research that has identified the role of 2-hydroxyphenyl acetic acid and 4-hydroxyphenyl acetic acid as defense compounds against *G. boninense* infection in oil palm.

Based on Table 3, glycogen and D-glucose are essential carbohydrate compounds found for severity indexes 3 and 4, respectively. Glycogen is essential to reserve polysaccharides for carbon and energy storage (similar function to starch), and D-glucose is also an energy source and the main starch component^56,57^. Based on these results, glycogen and D-glucose, which are strongly related to energy sources, are essential for host-pathogen interactions in BSR disease. Therefore, it can also be applied as a candidate biomarker for BSR disease.

The identification of this compound as a significant compound whose relative concentration increases in infected plants compared to healthy plants can be used as a basis for its application as a biomarker of BSR disease and a reference for further research on oil palm defense mechanisms against pathogenic fungal infections.

## Conclusion

In summary, metabolomic analysis using ^1^H-NMR successfully identified tentative biomarkers in *G. boninense*-infected oil palm stem tissue in 4 disease severity indices. These tentative biomarkers consisted of groups of chemical compounds, namely, organic acids, carbohydrates, compounds, organoheterocycles, nitrogenous organic compounds and benzene. On the other hand, pathway analysis successfully identified 5 signaling pathways that might be affected by BSR disease in oil palm stem tissue. The partial correlation-based analysis of the metabolic network also showed differences in metabolic network correlations in different severity indices. The tentative biomarkers obtained from the results of this study are to be used for diagnostic purposes. Likewise, the analysis of the oil palm pathway involved in this research is expected to serve as a reference for further studies, including genomic and transcriptomic studies. Further experiment are necessary with more samples to validate the proposed hypothesis

## Methods

### Sampling

Samples of oil palm stem tissue used in this study were collected from palm oil plantations of PT. Letawa, West Sulawesi, Indonesia (− 1° 19′ 53.84018″, 119° 25′ 41.96037″). The oil palm samples selected for this study were identified as infected by *Ganoderma boninense*. A total of 12 stem tissues of the 25-year-old oil palm plant with four different degrees of disease severity^58^ were sampled with 3 biological replicates for each disease severity index. The samples were taken on January 2021. The amount of biological replicates taken in this research was referred to previous research^58^ and based on the availability of samples that met the defined criteria that we used in this experiment. Oil palm with 4 disease severity indices is shown in Supplementary Figure 4. These indices contain of index 1 (healthy tree / control), index 2 (moderate healthy), index 3 (moderate severe), and index 4 (severe). Sampling of each oil palm was performed by taking stem tissue from three random locations for each plant. The stem tissue taken from any three locations with a height of 1–1.5 m from the ground is mixed together (bulking/mixing) to ensure that a plant's sampling is reasonably representative. The samples were then frozen using liquid nitrogen as a quenching step to stop the metabolic activity of the stem cells. The sample was then ground to form a powder and freeze dried to remove water content. Samples were stored at − 80 °C prior to further analysis.

### Metabolite Extraction

Sample extraction stages refer to a previous study^12^ with minor changes. A total of 150 mg stem tissue powder was extracted by sonication with an ultrasonic bath in 80% (v/v) methanol-water solution for 30 min at a temperature of 40 °C. This extraction step was repeated three times. The pooled supernatant was then filtered using filter paper (Whatman 125 mm) and evaporated using a rotary evaporator to obtain a crude solid extract. The crude extract was then stored at − 80 °C until further analysis.

### Nuclear magnetic resonance (NMR) measurement

A total of 150 mg of the crude extract was dissolved in 0.75 mL of deuterium solvent prepared from a 1:1 (v/v) mixture of methanol-d4 (99.8%; Merck) and KH_2_PO_4_ buffer (pH 6,0) in D_2_O (99.9%; Merck) with 0.1% (v/v) trimethylsilyl propionic acid d4 sodium salt (TSP) as an internal standard. The mixture was then vortexed for 1 min and sonicated for 20 min at room temperature. The solids were separated from the supernatant by centrifugation at 10,000 rpm for 10 min. A total of 0.6 ml of the supernatant was then transferred to the NMR tube for further analysis. J-resolved and 1H-NMR analyses were performed using an Agilent 500 MHz NMR spectrometer (Agilent Technologies Inc., Palo Alto, CA, USA). The measurements performed were 1D proton NMR measurements for 12 samples and 2D J-resolved NMR measurements for 4 selected samples for confirmation. In this measurement, a presaturation sequence is performed to eliminate residual water signals.

### Data analysis

^1^H NMR data processing was performed using a targeted metabolomics approach^59^. Free induction decay (FID) data were obtained from the measurements processed with Mestrenova software 8.0^60^. This stage includes the Fourier transform stage, phase and baseline correction^61^, and a TSP referencing a 0.0 chemical shift as a reference standard (Supplementary Figure 5). Data analysis was performed using the ASICS package^62^, a package in the R programming language environment that can perform metabolomics analysis on ^1^H NMR spectra to identify and quantify metabolites as referred to previous research^63,65^. This package could automatically identify and quantify metabolites in the NMR spectrum using a unique peak pattern (fingerprint)^59,62^. The phases of this process include importing CSV data obtained in the previous phase, normalization, alignment, exclusion of undesired areas where in this analysis are water (D_2_O) at chemical shift 4.5 to 5.1 ppm and methanol (CD_3_OD) at chemical shift of 3.33–3.38 ppm and identification of metabolites.

Multivariate statistical analysis was performed on a relative concentration table with MetaboAnalyst 5.0^64^. The variables were first normalized using the Pareto scaling method. In addition, a multivariate statistical analysis consisting of a principal component analysis (PCA), partial least squares discriminant analysis (PLS-DA), orthogonal and heatmap analysis was performed. PCA and PLS-DA analyses were performed to classify and distinguish samples based on multidimensional data generated from the analysis techniques performed. Variable determination was performed using the variable importance in projection (VIP) method, in which variables with a VIP value > 1.5 were further analyzed. Confirmation of metabolite identification was performed manually against multiple spectra.

### Metabolic pathway and network analysis

Metabolic pathway analysis was conducted to analyse the most important pathway chosen based on the *p* value ( <  0.05) and impact value score ( > 0.1)^37,65^. Pathway identification was performed using the Kyoto Encyclopedia of Genes and Genomes (KEGG) database^66^. Metabolic network analysis was conducted using the Debiased Sparse Partial Correlation algorithm that was integrated into the Network Analysis feature in MetaboAnalyst 5.0^27^.

### Plant research ethics declaration

We confirm that all methods in this study were carried out in accordance with relevant guidelines and legislation for plant research. Samples and data that were collected at palm oil plantations of PT. Letawa, West Sulawesi, Indonesia / PT. Astra Agro Lestari Tbk. not listed as endangered or threatened species. An ethical review by the Ministry of Environment and Forestry of The Republic of Indonesia was therefore not required.

## Supplementary Information

Supplementary Figure S1.

Supplementary Figure S2.

Supplementary Figure S3.

Supplementary Figure S4.

Supplementary Figure S5.

Supplementary Figure 1.

Supplementary Table 1.

SupplementaryTable 2.

Supplementary Information.

## Data Availability

All data generated or analysed during this study are included in this published article (and its supplementary information files).
