# Supplementary material for: 1H NMR metabolomics analysis of oil palm stem tissue infected by Ganoderma boninense based on field severity Indices
Source: Sci Rep. 2022 Dec 6;12:21087. doi: 10.1038/s41598-022-25450-5 (PMC9726981; doi:10.1038/s41598-022-25450-5)
Supplement: Supplementary file 1 — Supplementary Figure S1. [file 41598_2022_25450_MOESM1_ESM.pdf]

Supplementary Figures 1

(a) Index 1 and 2

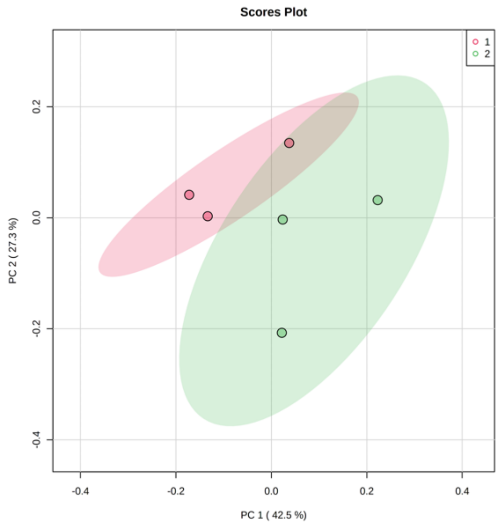

(b) Index 1 and 3

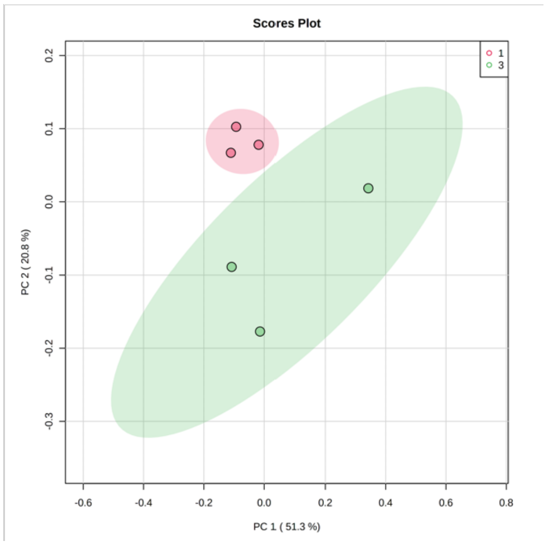

(c) Index 1 and 4

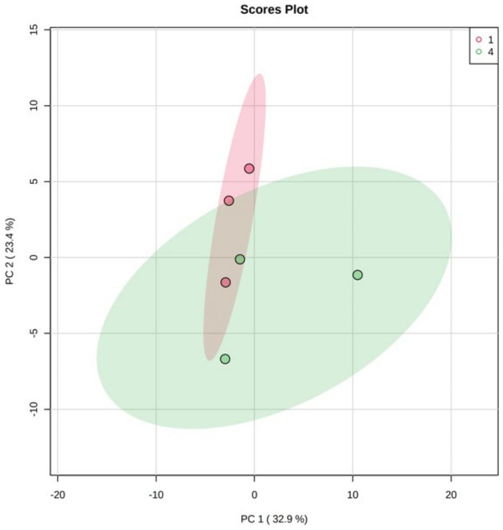

Figure S1. The results of 2D PCA visualization (a) indices 1 and 2, (b) indices 1 and 3, and (d) indices 1 and 4 to seedifferences in metabolite profiles in each severity conditions

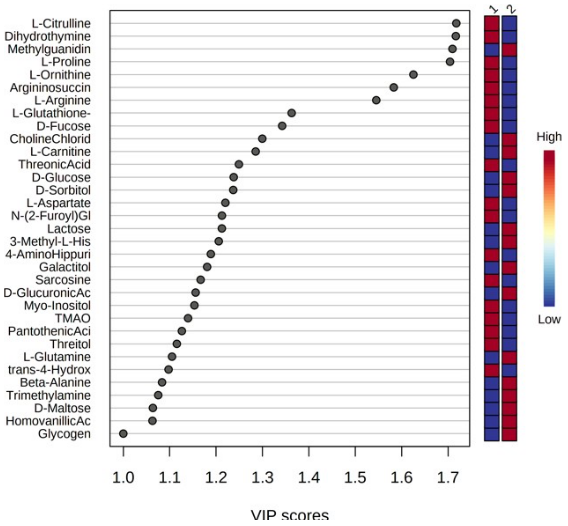

(a) Index 1 and 2

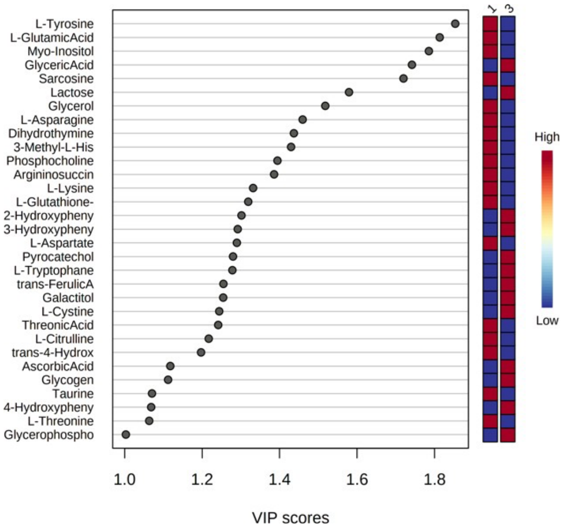

(b) Index 1 and 3

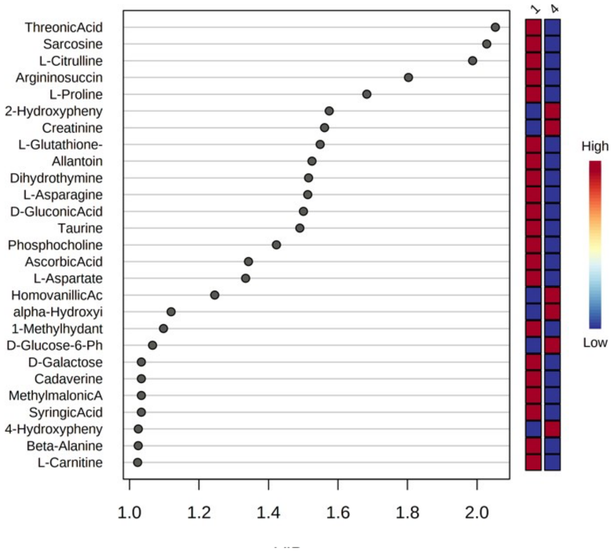

(c) Index 1 and 4

Figure S1. The results of the analysis of significant compounds using OPLS-DA analysis on (a) indexes 1 and 2, (b)indexes 1 and 3, and (c) indexes 1 and 4.
