# Supplementary material for: 1H NMR metabolomics analysis of oil palm stem tissue infected by Ganoderma boninense based on field severity Indices
Source: Sci Rep. 2022 Dec 6;12:21087. doi: 10.1038/s41598-022-25450-5 (PMC9726981; doi:10.1038/s41598-022-25450-5)
Supplement: Supplementary file 2 — Supplementary Figure S2. [file 41598_2022_25450_MOESM2_ESM.pdf]

Supplementary Figures 2

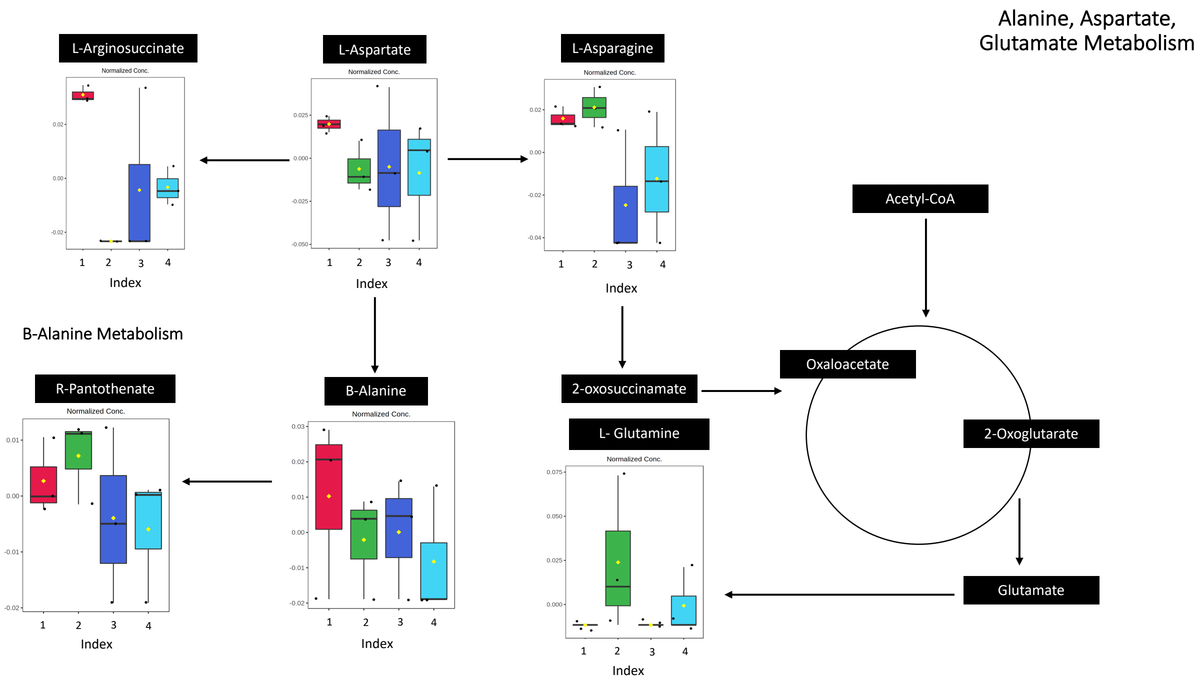

(a)

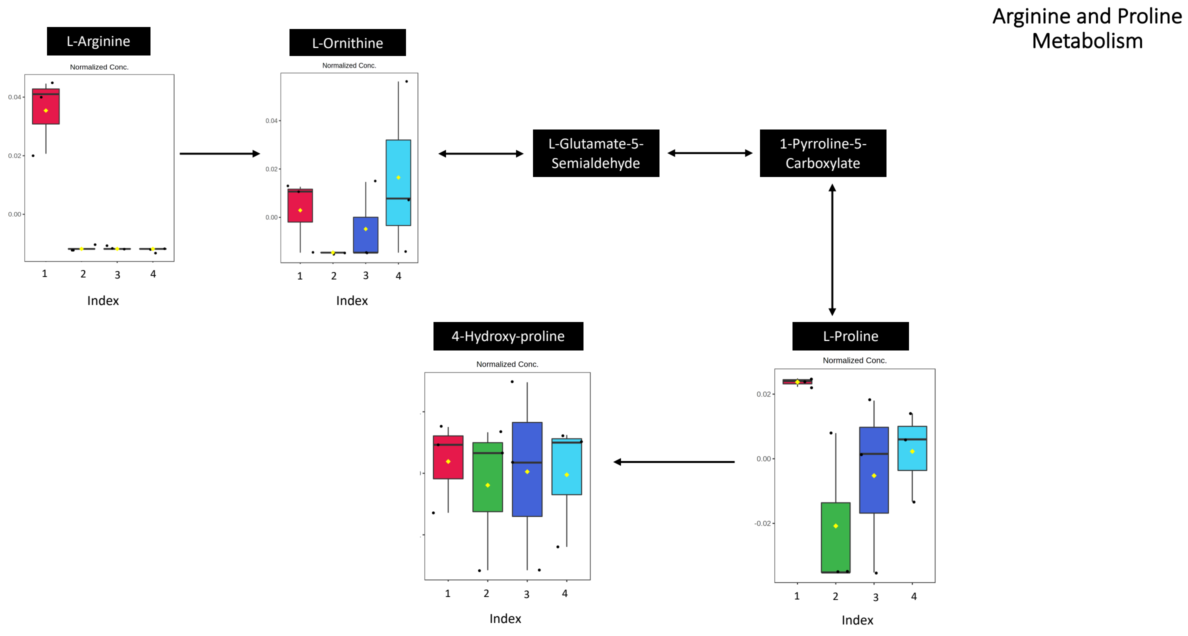

(b)

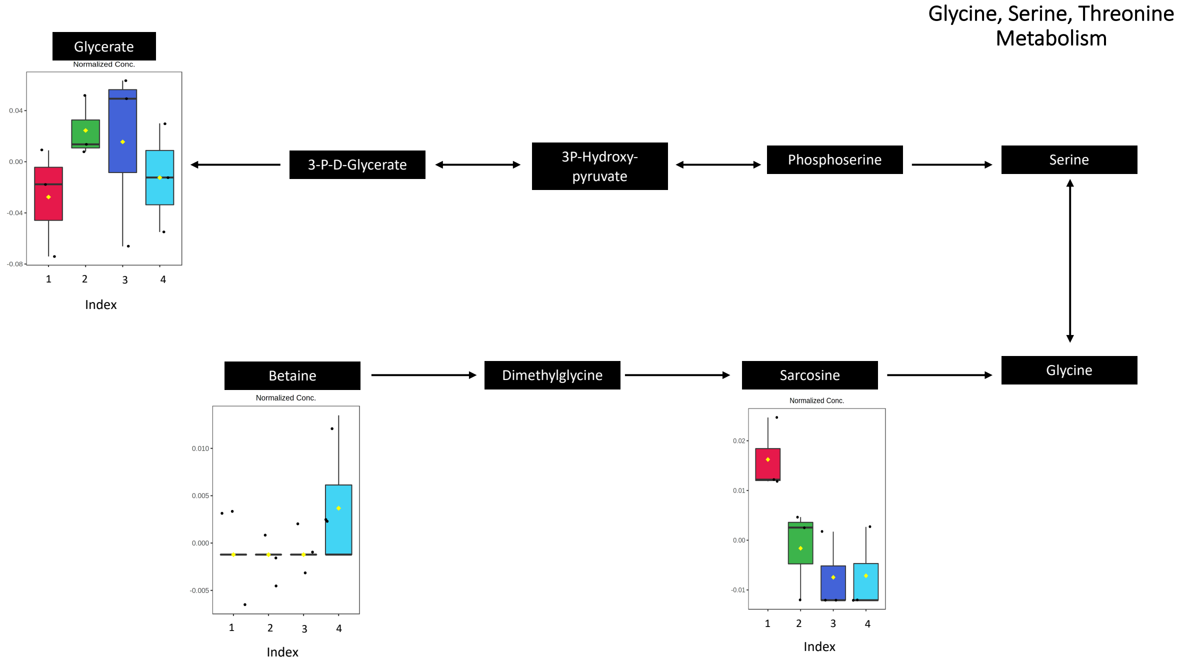

(c)

Figure S2. Schematic representation pathways those are potentially affected by BSR disease and relative concentrations of metabolites involved in (a) alanine, aspartate, glutamate metabolism and beta-alanine metabolism, (b) arginine and proline metabolism, and (c) glycine, serine, threonine metabolism.
