# Supplementary material for: 1H NMR metabolomics analysis of oil palm stem tissue infected by Ganoderma boninense based on field severity Indices
Source: Sci Rep. 2022 Dec 6;12:21087. doi: 10.1038/s41598-022-25450-5 (PMC9726981; doi:10.1038/s41598-022-25450-5)
Supplement: Supplementary file 3 — Supplementary Figure S3. [file 41598_2022_25450_MOESM3_ESM.pdf]

Supplementary Figures 3

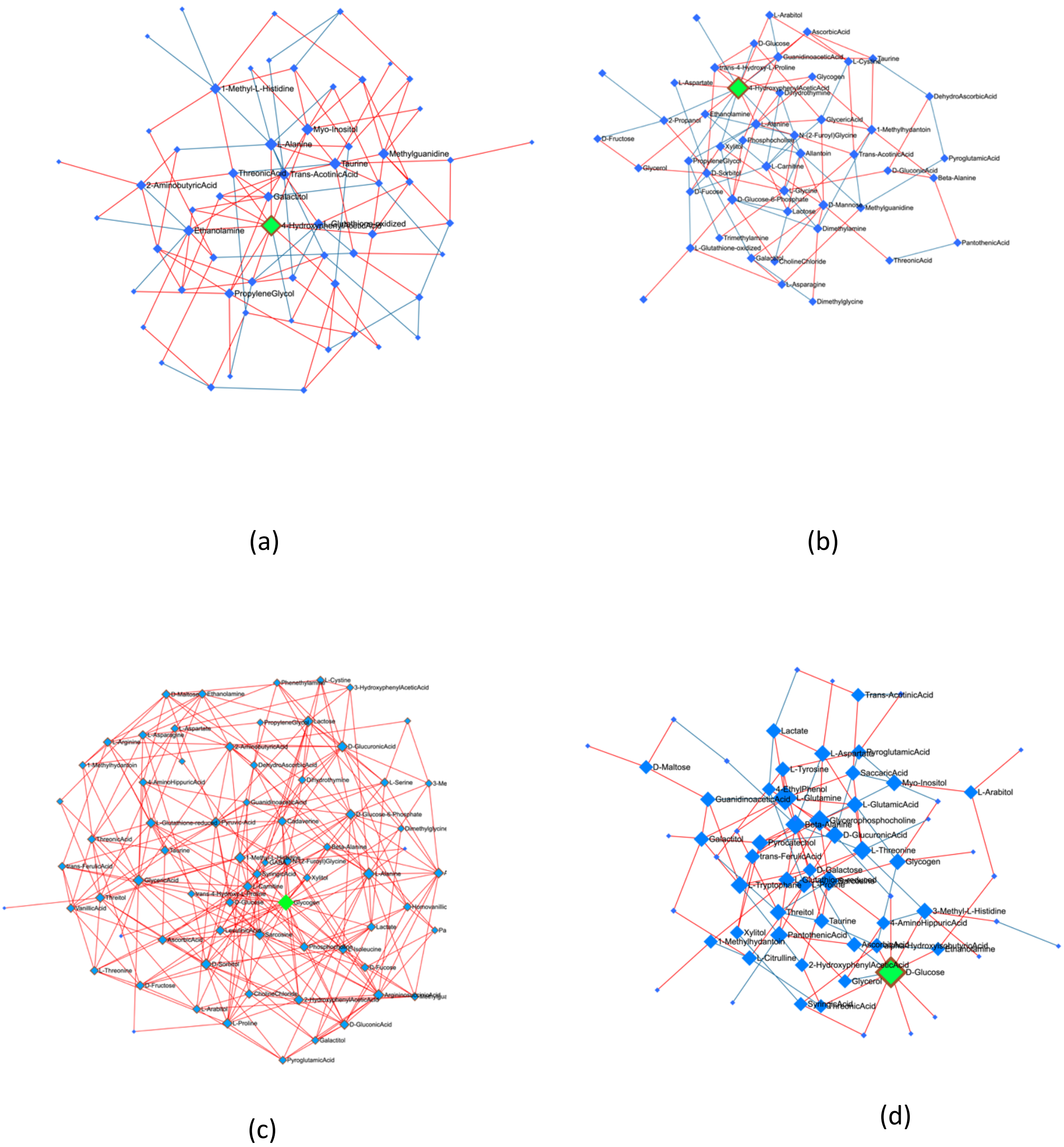

Figure S3. DSPC network analysis of (a) Index 1 (healthy) (b) Index 2 (moderate healthy) (d) Index 3 (moderate severe) (c) Index 4 (severe). Red edges indicate positive correlation and blue edges indicate negative correlation. Green nodes indicate nodes with highest nodes degree and betweenness central.
