# Supplementary material for: 1H NMR metabolomics analysis of oil palm stem tissue infected by Ganoderma boninense based on field severity Indices
Source: Sci Rep. 2022 Dec 6;12:21087. doi: 10.1038/s41598-022-25450-5 (PMC9726981; doi:10.1038/s41598-022-25450-5)
Supplement: Supplementary file 4 — Supplementary Figure S4. [file 41598_2022_25450_MOESM4_ESM.pdf]

Supplementary Figures 4

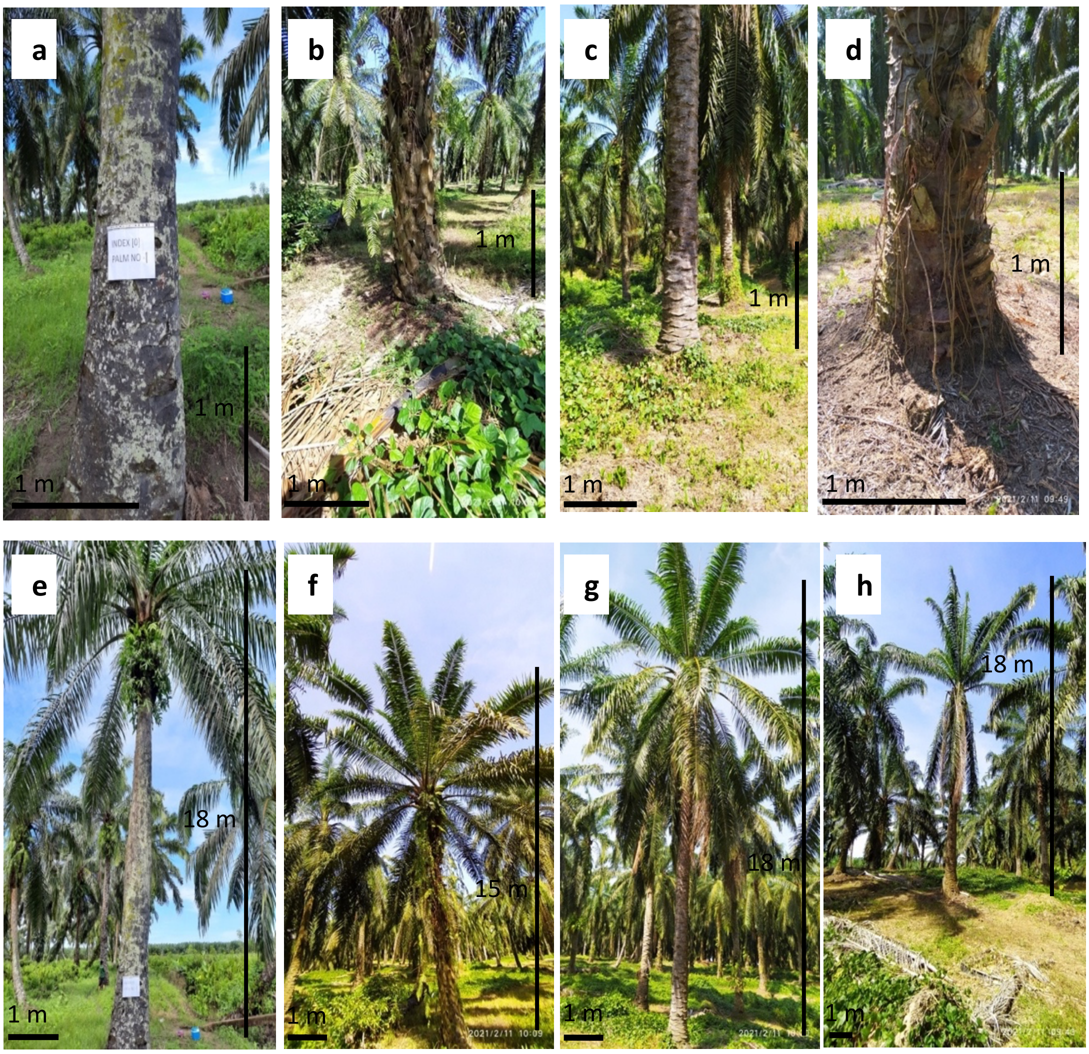

Figure S4. Oil palm samples used in this study. Stem of (a) Index 1 (Healthy), (b) Index 2 (Moderate Healthy), (c) Index 3 (Moderate Severe), and (d) Index 4 (Severe). And trees of (e) Index 1 (Healthy), (f) Index 2 (Moderate Healthy), (g) Index 3 (Moderate Severe), and (h) Index 4 (Severe). (Majid collection).
