# Supplementary material for: 1H NMR metabolomics analysis of oil palm stem tissue infected by Ganoderma boninense based on field severity Indices
Source: Sci Rep. 2022 Dec 6;12:21087. doi: 10.1038/s41598-022-25450-5 (PMC9726981; doi:10.1038/s41598-022-25450-5)
Supplement: Supplementary file 5 — Supplementary Figure S5. [file 41598_2022_25450_MOESM5_ESM.pdf]

Supplementary Figures 5

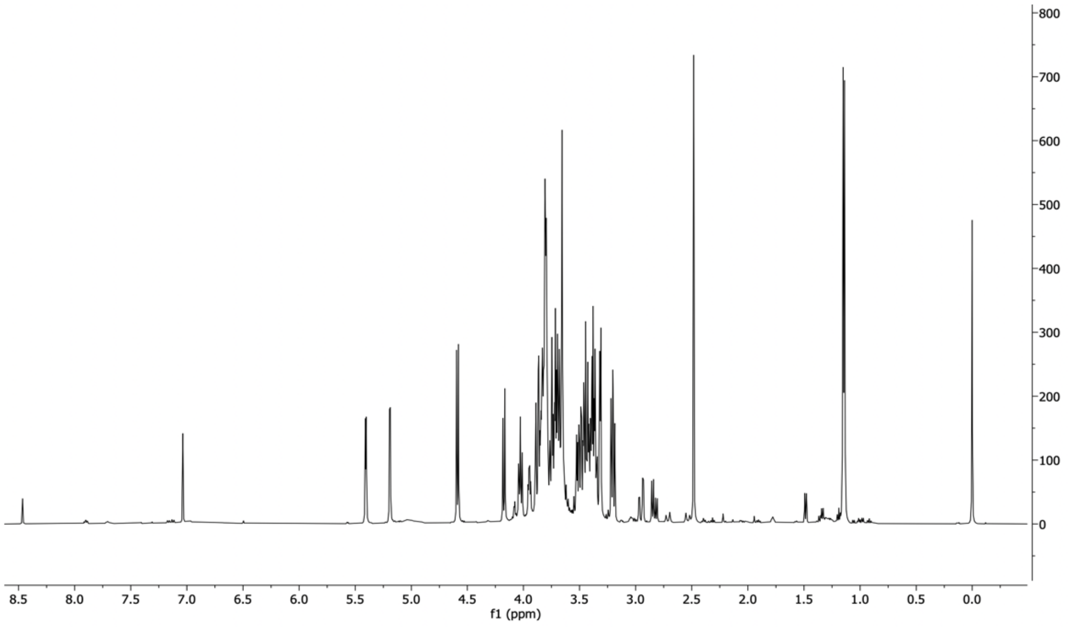

(a)

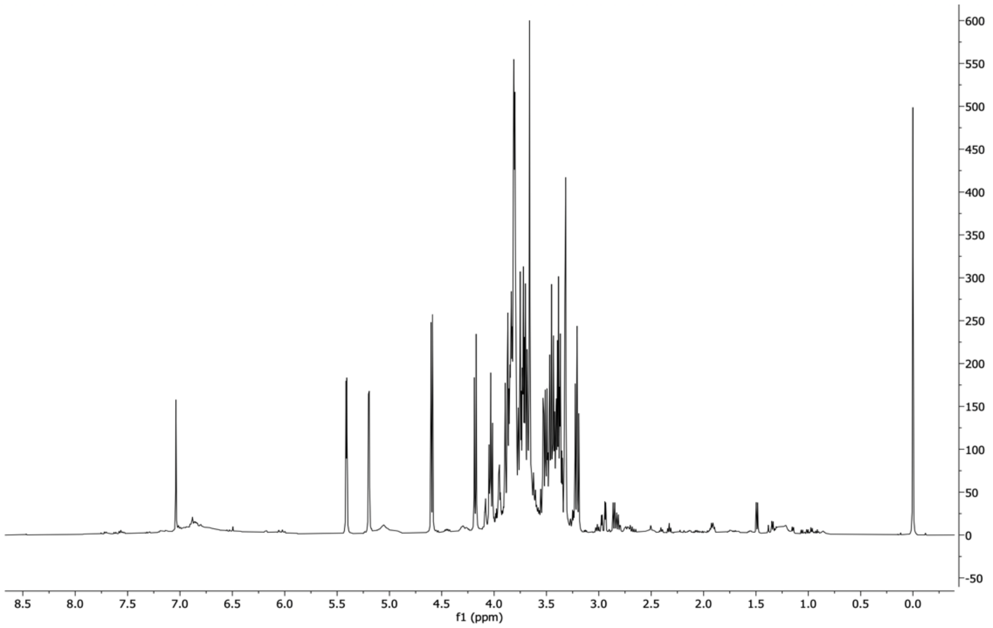

(b)

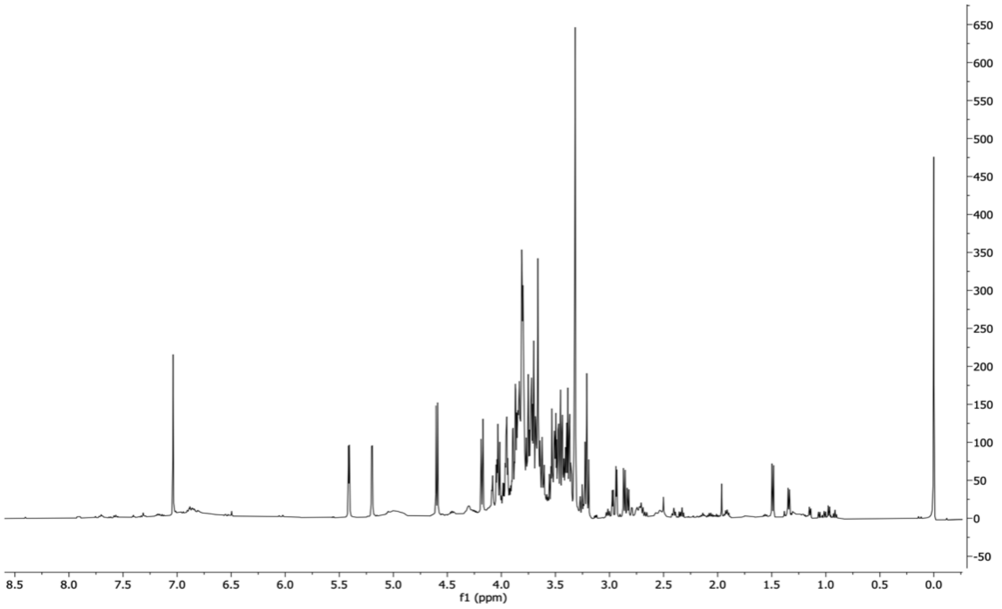

(c)

Figure S5. ^{1}H NMR spectra of (a) Index 2 (Moderate Healthy), (b) Index 3 (Moderate Severe), and (c) Index 4 (Severe).
