# Supplementary material for: 1H NMR metabolomics analysis of oil palm stem tissue infected by Ganoderma boninense based on field severity Indices
Source: Sci Rep. 2022 Dec 6;12:21087. doi: 10.1038/s41598-022-25450-5 (PMC9726981; doi:10.1038/s41598-022-25450-5)
Supplement: Supplementary file 6 — Supplementary Figure 1. [file 41598_2022_25450_MOESM6_ESM.pdf]

Supplementary Figure 1

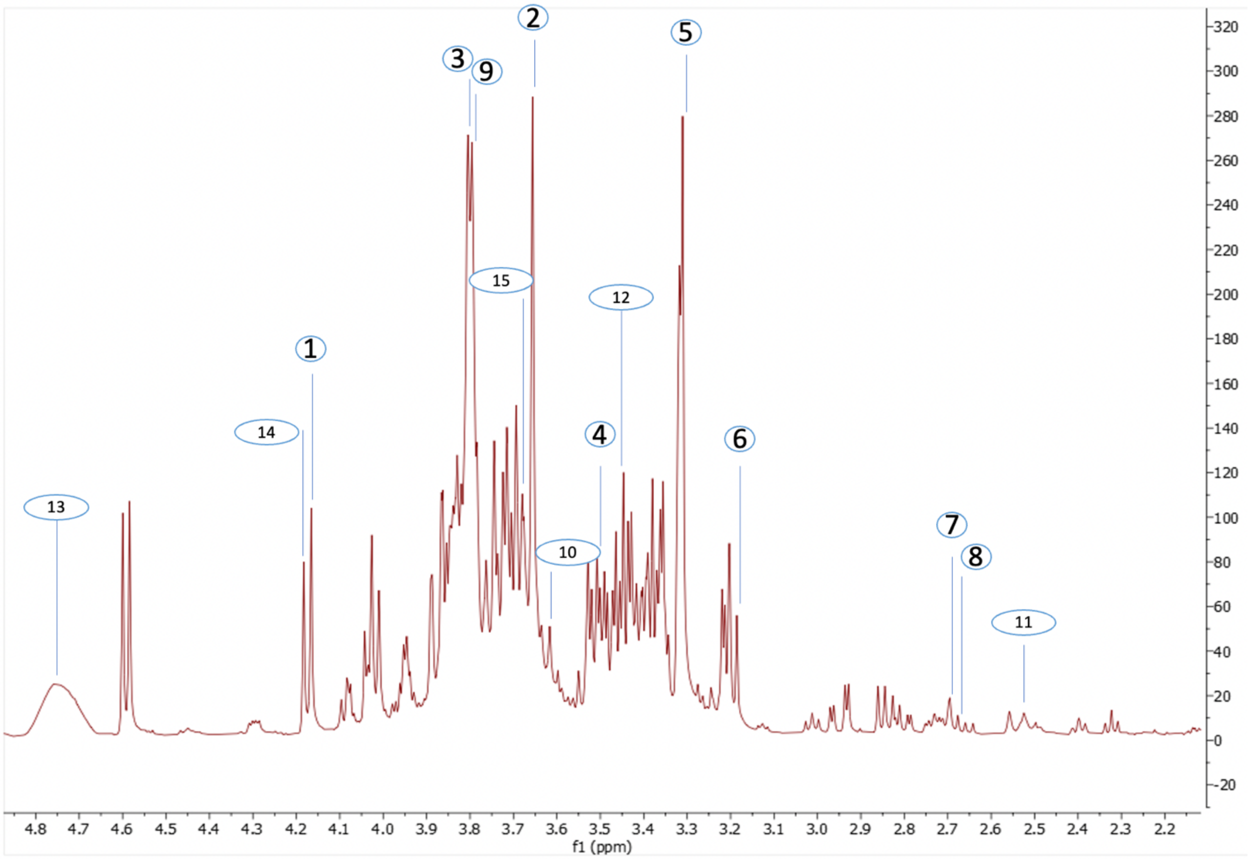

	Compounds	Index 1
1	DehydroAscorbicAcid	δ 3.638 - 3.81(m) , δ 4.17 - 4.18(d)
2	D-GluconicAcid	δ 3.69 - 4.09(m)
3	GuanidinoaceticAcid	δ 3.77(s)
4	D-Mannose	δ 3.52-3.54 (m)

5	D-Fructose	δ 3.35 - 4.2 (m)
6	L-Arabitol	δ 3.24 - 4.38 (m)
7	Taurine	δ 2.69-3.12 (m) , δ 7.43 - 7.65 (m)
8	Glycogen	δ 3.50 - 4.2(m) , δ 5.30 - 5.50(d)
9	Xylitol	δ 3.62 - 3.726(m) , δ 3.795 - 3.809(m)
10	Threitol	δ 3.652 - 3.712(m)
11	trans-4-Hydroxy-L-Proline	δ 2.00- 2.5(m) , δ 3.3-3.5(m), δ 4.2-4.6(m), 4.8 (s)
12	AscorbicAcid	δ 3.4 - 3.47(m) , δ 3.715-3.754(m), δ 4.72-4.857(m), 8.309(s), 11.034 (s)
13	Allantoin	δ 4.7 (s) , δ 4.825 (s), 5.375 (s)

14	L-Cystine	δ 3.13 - 3.188(m) , δ 4.295 - 4.405(d)
15	ThreonicAcid	δ 3.62(s) , δ 3.69(s),δ 3.98(s),δ 4.06(s)

Figure 1. ^{1}H NMR Spectra figures with complete annotation of several compounds
