# Supplementary material for: 1H NMR metabolomics analysis of oil palm stem tissue infected by Ganoderma boninense based on field severity Indices
Source: Sci Rep. 2022 Dec 6;12:21087. doi: 10.1038/s41598-022-25450-5 (PMC9726981; doi:10.1038/s41598-022-25450-5)
Supplement: Supplementary file 7 — Supplementary Table 1. [file 41598_2022_25450_MOESM7_ESM.pdf]

Supplementary Tables 1

Index 1	Index 2	Index 3	Index 4
DehydroAscorbicAcid	D-GluconicAcid	D-Glucose	D-Glucose
GuanidinoaceticAcid	DehydroAscorbicAcid	DehydroAscorbicAcid	GuanidinoaceticAcid
GlycericAcid	GuanidinoaceticAcid	GuanidinoaceticAcid	D-Mannose
D-GluconicAcid	D-Glucose	D-Mannose	GlycericAcid
AscorbicAcid	GlycericAcid	GlycericAcid	DehydroAscorbicAcid
D-Fructose	D-Mannose	Xylitol	Glycogen
Glycogen	D-GlucuronicAcid	Trans-AcotinicAcid	D-Glucose-6-Phosphate
trans-4-Hydroxy-L-Proline	ThreonicAcid	D-GlucuronicAcid	ThreonicAcid
D-Mannose	Glycogen	D-Fructose	trans-4-Hydroxy-L-Proline
L-Arabitol	Xylitol	Threitol	Xylitol
Allantoin	trans-4-Hydroxy-L-Proline	D-Sorbitol	L-Arabitol
Taurine	D-Fructose	AscorbicAcid	Taurine
Xylitol	D-Maltose	Glycogen	L-Asparagine
L-Cystine	L-Cystine	L-Arabitol	L-Cystine
L-Aspartate	L-Aspartate	trans-4-Hydroxy-L-Proline	D-Maltose
Trans-AcotinicAcid	Allantoin	ArgininosuccinicAcid	D-GluconicAcid
L-Asparagine	L-Arabitol	ThreonicAcid	D-Fructose
D-Sorbitol	Taurine	L-Cystine	Threitol
ThreonicAcid	L-Asparagine	L-Asparagine	L-Citrulline
Glycerol	4-HydroxyphenylAceticAcid	Taurine	AscorbicAcid
L-Citrulline	Lactose	L-Aspartate	Galactitol
L-Arginine	Ethanolamine	Lactose	Glycerol
ArgininosuccinicAcid	PyroglutamicAcid	Ethanolamine	L-Tryptophane
L-Proline	Trans-AcotinicAcid	L-Glutathione-reduced	L-Aspartate
Threitol	Beta-Alanine	CholineChloride	L-Glutathione-oxidized
HomovanillicAcid	Glycerol	L-Proline	Lactose
PropyleneGlycol	D-Sorbitol	L-Alanine	2-HydroxyphenylAceticAcid
4-HydroxyphenylAceticAcid	L-Alanine	PyroglutamicAcid	L-Ornithine

PyroglutamicAcid
L-Alanine
L-Glutathione-oxidized
Myo-Inositol
Ethanolamine
2-AminobutyricAcid
1-Methyl-L-Histidine
L-Threonine
D-Fucose
Beta-Alanine
CholineChloride
Dihydrothymine
L-Glycine
3-Methyl-L-Histidine
PantothenicAcid
Methylguanidine
Sarcosine
Phosphocholine
1-Methylhydantoin
Dimethylglycine
Glycerophosphocholine
D-Glucose
D-Maltose
D-Glucose-6-Phosphate
L-Ornithine
N-(2-Furoyl)Glycine
SyringicAcid
Dimethylamine
D-GlucuronicAcid
D-Galactose
L-Tryptophane
3-HydroxyphenylAceticAcid

2-HydroxyphenylAceticAcid
D-Fucose
N-(2-Furoyl)Glycine
Methylguanidine
CholineChloride
3-HydroxyphenylAceticAcid
L-Glycine
L-Carnitine
PropyleneGlycol
1-Methylhydantoin
3-Methyl-L-Histidine
PantothenicAcid
Dihydrothymine
Dimethylglycine
SyringicAcid
Dimethylamine
AscorbicAcid
Galactitol
D-Glucose-6-Phosphate
L-Glutathione-oxidized
Phosphocholine

2-HydroxyphenylAceticAcid
L-Tryptophane
D-GluconicAcid
D-Maltose
HomovanillicAcid
MalicAcid
3-HydroxyphenylAceticAcid
Glycerol
L-Glutathione-oxidized
D-Fucose
Phosphocholine
L-Arginine
Allantoin
D-Glucose-6-Phosphate
Beta-Alanine
2-AminobutyricAcid
1-Methyl-L-Histidine
4-HydroxyphenylAceticAcid
Methylguanidine
4-AminoHippuricAcid
PropyleneGlycol
1-Methylhydantoin
Cadaverine
L-Threonine
Dimethylglycine
PantothenicAcid
Dihydrothymine
Lactate
Sarcosine
L-Isoleucine
SyringicAcid
L-Carnitine

Ethanolamine
MalicAcid
ArgininosuccinicAcid
4-HydroxyphenylAceticAcid
L-Alanine
D-Fucose
PyroglutamicAcid
Beta-Alanine
L-Proline
HomovanillicAcid
Dihydrothymine
Trans-AcotinicAcid
PropyleneGlycol
3-Methyl-L-Histidine
Methylguanidine
PantothenicAcid
CholineChloride
1-Methyl-L-Histidine
SyringicAcid
Dimethylamine
L-Carnitine
D-Sorbitol
D-GlucuronicAcid
L-Threonine
Lactate
D-Galactose
3-HydroxyphenylAceticAcid
L-Glutathione-reduced
Myo-Inositol
Glycerophosphocholine
2-AminobutyricAcid
1-Methylhydantoin

L-Glutathione-reduced
Galactitol
2-HydroxyphenylAceticAcid
Cadaverine

N-(2-Furoyl)Glycine
Galactitol
trans-FerulicAcid
3-Methyl-L-Histidine

4-AminoHippuricAcid
trans-FerulicAcid
L-Isoleucine
Sarcosine

Table 1. Exclusive metabolites for Index 1, Index 2, Index 3, and Index 4
