# Supplementary material for: 1H NMR metabolomics analysis of oil palm stem tissue infected by Ganoderma boninense based on field severity Indices
Source: Sci Rep. 2022 Dec 6;12:21087. doi: 10.1038/s41598-022-25450-5 (PMC9726981; doi:10.1038/s41598-022-25450-5)
Supplement: Supplementary file 8 — SupplementaryTable 2. [file 41598_2022_25450_MOESM8_ESM.pdf]

Supplementary Table 2

Compounds	Index 1_A	Index 1_B	Index 1_C	Index 2_A	Index 2_B	Index 2_C	Index 3_A	Index 3_B	Index 3_C	Index 4_A	Index 4_B	Index 4_C
DehydroAscorbic Acid	7E+14	1E+14	8E+14	9E+14	8E+14	1E+14	1E+14	8E+14	6E+14	8E+14	0	5E+14
GlycericAcid	6E+13	5E+14	3E+14	6E+14	7E+14	8E+14	8E+14	3E+14	9E+14	5E+14	4E+14	7E+14
D-GluconicAcid	6E+14	1E+15	6E+14	7E+14	7E+14	1E+15	6E+14	7E+14	4E+14	4E+14	4E+14	2E+13
GuanidinoaceticAcid	6E+14	1E+15	4E+14	6E+14	7E+14	1E+13	9E+13	6E+13	5E+14	5E+14	5E+14	3E+14
D-Mannose	6E+14	6E+14	5E+14	7E+14	8E+14	9E+14	1E+15	3E+14	4E+14	6E+14	2E+14	4E+14
D-Fructose	5E+13	3E+13	4E+14	4E+14	3E+13	5E+13	4E+14	3E+14	3E+14	3E+14	1E+14	3E+14
L-Arabitol	4E+14	1E+14	2E+14	9E+14	3E+14	2E+14	4E+14	0	0	1E+14	0	2E+14
Taurine	4E+14	3E+14	2E+14	2E+14	2E+14	3E+14	4E+14	0	1E+14	2E+14	9E+14	1E+13
Glycogen	3E+14	5E+14	3E+14	3E+14	3E+14	5E+14	3E+14	3E+14	3E+14	2E+14	4E+14	3E+14
Xylitol	3E+14	5E+14	3E+14	3E+14	5E+13	6E+14	5E+14	2E+14	3E+14	3E+14	3E+13	2E+14
Threitol	3E+14	0	2E+14	0	3E+14	0	4E+13	0	0	2E+14	0	1E+14
trans-4-Hydroxy-L-Proline	3E+14	1E+14	3E+14	3E+14	0	2E+14	4E+14	0	2E+14	3E+14	6E+14	3E+14
AscorbicAcid	3E+14	5E+14	4E+12	1E+14	2E+14	3E+14	2E+14	3E+14	3E+14	1E+14	3E+14	3E+14
Allantoin	3E+14	9E+14	4E+14	2E+14	2E+11	5E+14	3E+13	4E+14	0	0	0	0
L-Cystine	2E+14	2E+14	2E+14	2E+14	3E+14	2E+14	3E+14	1E+14	2E+14	2E+14	7E+14	2E+14
ThreonicAcid	2E+14	5E+14	2E+14	3E+14	3E+14	3E+14	3E+14	0	0	1E+14	2E+14	0
D-Sorbitol	2E+14	0	0	1E+14	4E+14	5E+14	7E+14	0	4E+14	3E+14	0	2E+14
L-Aspartate	2E+14	2E+14	2E+14	2E+14	1E+14	1E+14	3E+14	0	1E+14	2E+14	0	2E+13
Glycerol	2E+14	1E+14	1E+14	1E+14	1E+14	2E+14	1E+14	7E+13	0	9E+14	2E+14	1E+14
L-Asparagine	2E+14	2E+14	2E+13	2E+13	2E+14	2E+14	2E+14	0	0	1E+15	0	2E+14

Trans-AcotinicAcid	2E+14	2E+14	2E+13	2E+14	0	3E+14	5E+14	1E+14	2E+14	3E+14	2E+14	1E+11
L-Proline	1E+14	2E+14	2E+13	0	0	1E+13	1E+13	0	1E+14	1E+14	7E+14	1E+14
Ethanolamine	1E+14	2E+14	1E+15	1E+14	1E+14	2E+14	2E+14	0	1E+14	1E+14	1E+15	9E+14
ArgininosuccinicAcid	1E+13	2E+14	1E+14	0	0	0	2E+14	0	0	8E+14	6E+14	4E+13
L-Arginine	1E+14	9E+13	1E+14	0	0	0	0	0	0	0	0	0
L-Tryptophane	1E+14	0	0	0	0	0	1E+14	1E+14	0	1E+13	0	1E+14
4-HydroxyphenylAceticAcid	1E+14	1E+14	1E+15	7E+14	2E+14	2E+14	1E+14	5E+14	2E+14	8E+14	0	1E+14
L-Glutathione-oxidized	1E+14	2E+13	8E+14	0	0	1E+14	7E+14	0	3E+14	5E+14	1E+14	0
Myo-Inositol	1E+14	0	1E+14	0	0	0	0	0	0	0	0	6E+14
L-Citrulline	1E+13	1E+14	1E+14	0	0	0	1E+15	0	0	0	1E+14	0
PyroglutamicAcid	1E+14	1E+14	7E+14	1E+15	1E+15	9E+14	1E+14	0	5E+14	0	2E+14	8E+14
D-Fucose	9E+14	1E+14	8E+14	8E+14	8E+14	9E+14	1E+13	0	8E+14	9E+14	9E+14	6E+14
L-Alanine	9E+14	9E+14	1E+14	7E+14	9E+14	7E+14	1E+14	5E+14	5E+14	8E+14	8E+14	9E+14
2-AminobutyricAcid	9E+14	0	0	0	0	0	0	0	0	0	0	0
PropyleneGlycol	8E+13	3E+14	4E+13	3E+14	7E+14	5E+14	4E+14	3E+14	0	3E+14	2E+14	3E+14
L-GlutamicAcid	8E+14	0	1E+15	0	0	0	0	0	0	0	1E+14	0
L-Threonine	7E+14	0	4E+13	0	0	0	0	0	0	0	2E+14	6E+14
L-Ornithine	7E+14	7E+14	0	0	0	0	8E+14	0	0	6E+14	2E+14	0
L-Leucine	6E+14	0	0	0	0	0	0	0	0	0	0	0
3-Methyl-L-Histidine	6E+14	0	3E+14	5E+14	5E+14	1E+14	0	0	2E+14	0	6E+13	2E+14
CholineChloride	6E+14	2E+14	3E+13	4E+14	8E+14	1E+14	1E+14	1E+14	8E+14	5E+13	9E+08	6E+14

4-AminoHippuricAcid	5E+14	0	6E+13	0	0	0	0	6E+14	0	0	3E+14	5E+14
Dihydrothymine	5E+14	6E+14	6E+14	0	0	0	0	0	3E+14	0	4E+14	3E+12
L-Carnitine	4E+14	3E+14	2E+14	4E+14	7E+14	5E+14	1E+13	0	3E+14	5E+14	0	2E+13
L-Glycine	4E+14	0	0	0	0	0	0	0	0	0	0	0
1-Methylhydantoin	4E+14	4E+13	6E+14	4E+13	5E+13	2E+14	6E+14	3E+14	2E+14	3E+14	0	4E+14
Citrate	4E+14	7E+14	0	0	0	4E+14	0	0	0	0	0	0
1-Methyl-L-Histidine	4E+14	0	4E+14	4E+14	0	8E+14	1E+14	0	0	4E+14	0	3E+14
PantothenicAcid	4E+14	2E+14	2E+14	2E+12	4E+12	4E+14	4E+12	0	2E+14	3E+14	0	2E+13
Sarcosine	3E+14	3E+14	5E+14	2E+14	0	2E+14	2E+14	0	0	0	0	2E+14
Lactate	3E+14	4E+14	3E+14	0	4E+14	0	0	0	0	0	8E+14	0
L-Tyrosine	3E+14	0	3E+14	0	3E+12	0	0	0	0	0	0	0
HomovanillicAcid	3E+14	0	0	0	0	0	0	0	0	0	0	0
Methylguanidine	2E+13	5E+14	0	0	3E+14	4E+14	7E+14	0	0	2E+13	0	0
2-Propanol	2E+14	0	0	0	2E+14	0	0	0	0	0	1E+14	0
Dimethylglycine	2E+14	2E+14	0	3E+14	3E+14	3E+14	3E+14	0	1E+14	1E+14	0	2E+14
Glycerophosphocholine	1E+10	1E+10	8E+09	7E+09	0	0	1E+13	0	1E+14	0	0	1E+14
D-Glucose	0	8E+14	0	7E+14	8E+14	1E+14	1E+14	0	7E+14	7E+14	3E+14	0
D-Glucose-6-Phosphate	0	4E+14	0	2E+14	1E+14	2E+14	4E+14	0	2E+14	3E+14	2E+14	0
D-Maltose	0	2E+14	0	5E+13	1E+14	7E+14	1E+14	0	1E+14	1E+14	8E+14	0
MalicAcid	0	1E+14	0	0	0	0	9E+14	0	0	0	0	0
2-HydroxyphenylAceticAcid	0	9E+12	0	1E+14	0	0	1E+13	2E+14	1E+14	1E+15	8E+14	1E+14
Beta-Alanine	0	8E+14	1E+15	5E+14	0	6E+13	7E+14	0	5E+14	7E+14	0	0

L-Lysine	0	8E+14	0	0	0	0	9E+14	0	0	0	0	9E+13
2-AminoAdipicAcid	0	8E+14	0	0	0	0	7E+14	0	0	0	0	0
Galactitol	0	7E+14	7E+14	0	1E+14	0	2E+14	0	9E+14	1E+14	0	5E+14
3-HydroxyphenylAceticAcid	0	6E+14	0	7E+14	0	0	1E+14	4E+14	7E+14	0	4E+13	0
L-Glutathione-reduced	0	5E+14	9E+14	0	0	8E+14	8E+14	0	0	5E+14	0	1E+14
Dimethylamine	0	2E+14	0	0	0	2E+14	0	0	0	1E+14	0	0
Phosphocholine	0	1E+14	0	0	0	1E+10	0	0	6E+09	0	2E+14	0
N-(2-Furoyl)Glycine	0	0	6E+14	0	0	0	0	0	0	0	0	0
4-EthylPhenol	0	0	3E+14	0	1E+14	0	0	0	0	0	1E+14	0
D-GlucuronicAcid	0	0	0	4E+14	0	5E+14	0	0	2E+14	0	4E+13	0
Lactose	0	0	0	9E+14	8E+14	0	2E+14	1E+14	8E+14	9E+13	0	0
Levoglucosan	0	0	0	9E+14	0	1E+14	0	0	0	0	0	0
L-Glutamine	0	0	0	7E+14	2E+14	0	0	0	0	0	1E+15	0
Formate	0	0	0	0	6E+14	0	0	0	0	0	0	0
GABA	0	0	0	0	4E+13	0	7E+14	0	0	0	0	0
7-Methylxanthine	0	0	0	0	0	0	6E+14	0	0	0	0	0
Hypotaurine	0	0	0	0	0	0	5E+14	0	0	0	0	0
beta-Hydroxyisovaleric Acid	0	0	0	0	0	0	1E+14	0	0	0	0	0
D-Galactose	0	0	0	0	0	0	0	3E+14	0	0	0	0
L-Serine	0	0	0	0	0	0	0	1E+15	0	0	0	0
trans-FerulicAcid	0	0	0	0	0	0	0	3E+14	0	0	0	2E+14
VanillicAcid	0	0	0	0	0	0	0	2E+14	0	0	0	0

SyringicAcid	0	0	0	0	0	0	0	2E+14	0	0	0	1E+13
Pyrocatechol	0	0	0	0	0	0	0	0	6E+14	7E+14	6E+14	0
alpha-Hydroxyisobutyric Acid	0	0	0	0	0	0	0	0	0	0	9E+14	2E+14
GlycolicAcid	0	0	0	0	0	0	0	0	0	0	0	7E+14
Betaine	0	0	0	0	0	0	0	0	0	0	0	8E+09
